# Substituted Pyrazoles and Their Heteroannulated Analogs—Recent Syntheses and Biological Activities

**DOI:** 10.3390/molecules26164995

**Published:** 2021-08-18

**Authors:** Mohamed Ramadan, Ashraf A. Aly, Lamiaa E. Abd El-Haleem, Mohammed B. Alshammari, Stefan Bräse

**Affiliations:** 1 Organic Chemistry Department, Faculty of Pharmacy, Al-Azhar University, Assiut Branch, Assuit 71524, Egypt; elbashamohammed@yahoo.com; 2 Chemistry Department, Faculty of Science, Minia University, El-Minia 61519, Egypt; lamiaaelsayed2013@yahoo.com; 3 Chemistry Department, College of Sciences and Humanities, Prince Sattam Bin Abdulaziz University, P.O. Box 83, Al-Kharij 11942, Saudi Arabia; m.alshammari@psau.edu.sa; 4 Institute of Organic Chemistry, Karlsruhe Institute of Technology, 76131 Karlsruhe, Germany; 5 Institute of Biological and Chemical Systems (IBCS-FMS), Karlsruhe Institute of Technology, 76344 Eggenstein-Leopoldshafen, Germany

**Keywords:** pyrazole, heteroannulated, synthesis, reactions, biological activity

## Abstract

Pyrazoles are considered privileged scaffolds in medicinal chemistry. Previous reviews have discussed the importance of pyrazoles and their biological activities; however, few have dealt with the chemistry and the biology of heteroannulated derivatives. Therefore, we focused our attention on recent topics, up until 2020, for the synthesis of pyrazoles, their heteroannulated derivatives, and their applications as biologically active moieties. Moreover, we focused on traditional procedures used in the synthesis of pyrazoles.

## 1. Introduction

**Figure 1:**
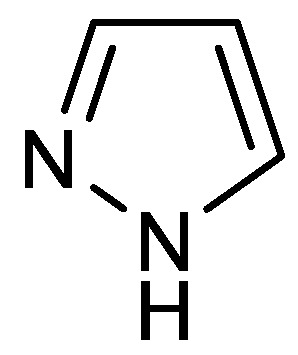
Structure of pyrazole.

Pyrazoles consist of two nitrogen atoms adjacent to three carbon atoms in a five-membered aromatic ring structure (Figure 1). Due to the broad spectrum of biological activities, the pyrazole ring is considered an interesting class in drug discovery [1].

**Figure 2:**
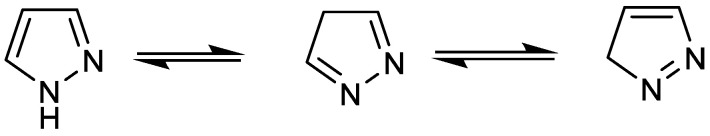
Tautomeric forms of unsubstituted pyrazole.

Unsubstituted pyrazole can be represented in three tautomeric forms [2] (Figure 2).

**Figure 3:**
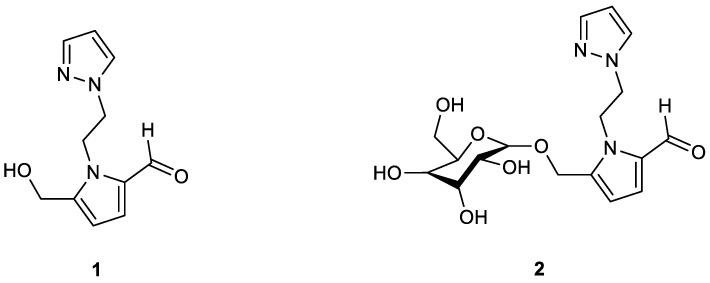
Structure of isolated pyrazoles of watermelon seeds.

Interestingly, pyrazoles as a class of azoles, are found in naturally occurring compounds. Kikuchi et al. [3] reported on two compounds, 1-[2-(5-hydroxymethyl-1*H*-pyrrole-2-carbaldehyde-1-yl)ethyl]-1*H*-pyrazole (**1**) and 1-({[5-(α-d-galactopyranosyloxy)methyl]-1*H*-pyrrole-2-carbaldehyde-1-yl}-ethyl)-1*H*-pyrazole (**2**), which were isolated from an extract of watermelon seeds (Figure 3). Pyrazoles also display innumerable chemical, biological, agrochemical, and pharmacological properties [4]. Moreover, a large number of structurally diverse natural compounds containing azole nucleus constitute an important class of biologically active heterocycles that are gaining more attention in the field of medicinal chemistry [5].

Many pyrazoles have shown luminescent and fluorescent agents. Some of these compounds have important applications in material chemistry [6] and as brightening agents [7]. Others exhibit solvatochromic [8] and electroluminescence [9] properties. Moreover, some pyrazoles act as semiconductors [10], liquid crystals [11], and organic light-emitting diodes [12].

In biological aspects, pyrazoles are known to exhibit antibacterial [13], anticancer [14,15], anti-tubercular [16], anti-inflammatory [17,18], antidepressant [19,20], antifungal [21], anxiolytic [22], anti-AIDS [23], and anti-malarial activities [24]. Pyrazoles also exhibit promising antioxidant activities [25], analgesic properties [26], they bind to estrogen receptors [27], they have neuroprotective properties [28], have the capability of binding to the monoamine oxidase enzyme [29], they have antihepatotoxicity properties [30], antileishmanial properties [31], antiproliferative properties [32], are preferred for tissue non-specific alkaline phosphatase inhibitor inhibition [33], act as cyclin-dependent kinase inhibitors [34], have anti-hyperglycemic properties [35], anti-nitric oxide synthases (NOSs) [36], have immunosuppressant properties [37], and demonstrate insecticidal activities [38]. 

**Figure 4:**
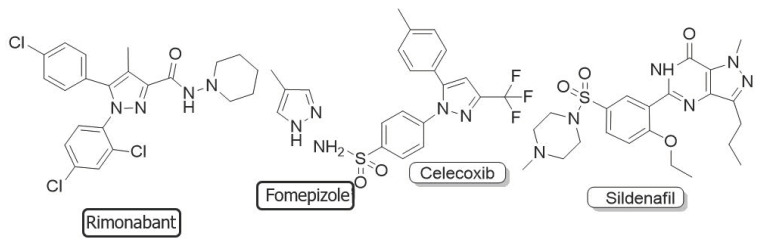
Drug molecules containing pyrazole scaffolds.

Pyrazoles are frequently observed as bioactive components in commercially available medicines. For example, **rimonabant** is a cannabinoid ligand and is used for treating obesity; **fomepizole** prevents alcohol dehydrogenase, **celecoxib** is a nonsteroidal anti-inflammatory drug (NSAID), specifically, a COX-2 inhibitor, which relieves pain and inflammation, and **sildenafil** is a PDE_5_ inhibitor used in the treatment of erectile dysfunction [39] (Figure 4).

**Scheme 1:**
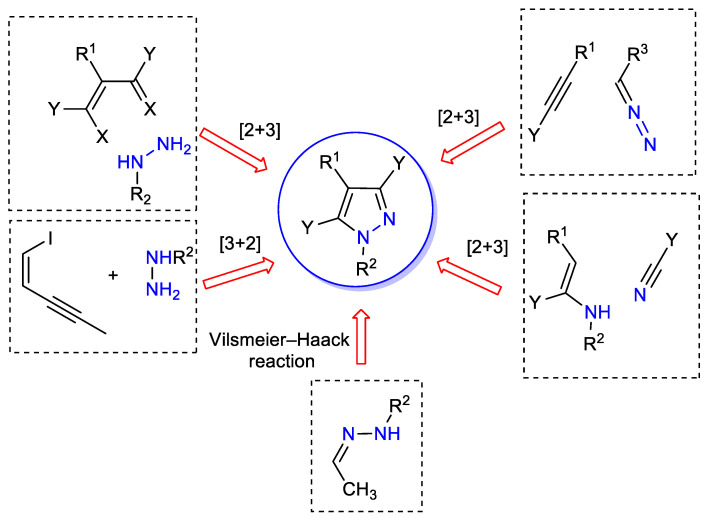
The general approach to form pyrazole derivatives.

This review summarizes the updated methods (until the end of 2020) that are generally used to prepare substituted pyrazoles and their heteroannulated pyrazoles and sheds light on their biological activities. Different approaches can be considered for synthesizing pyrazoles, such as 2 + 2 + 1, 2 + 3, 4 + 1, 6 − 1, etc. (Scheme 1).

In addition, these methods can be combined with metal-catalyzed, organo-catalyzed, flow chemistry, and other methods. In this context, many methods address atom economy (“green”) and multi-component reactions.

## 2. Synthesis of Pyrazoles

### 2.1. Cyclocondensation of Hydrazines

#### 2.1.1. Cyclocondensation of Hydrazines with 1,3-Dicarbonyl Compounds

**Scheme 2:**
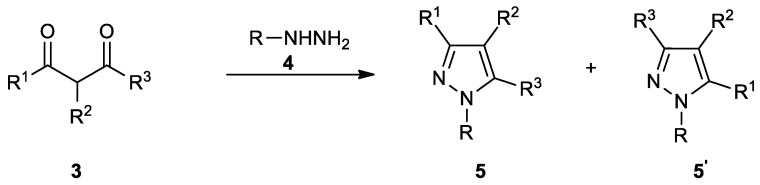
Knorr synthesis of pyrazoles **5** and **5′**.

Cyclocondensation of 1,3-dicarbonyl compounds **3** with substituted hydrazines **4** gave the corresponding substituted pyrazoles regioisomers **5** and **5′** (Scheme 2) in different yield percentages depending on the electronic effects, such as the inductive (electron or withdrawing character) and the steric factors of both substituents R^1^ and R^3^ (R^1^ and R^3^ are unequal). For example, if R^1^ constitutes an aryl group and R^3^ constitutes an alkyl substituent, the reaction proceeds, under conventional conditions, to give the regioisomer **5** as the major product, whereas **5′** is formed in traces. The selectivity obtained is of the order of 98:2 (i.e., R^1^ = Ar and R^3^ = CH_3_) [40].

**Scheme 3:**
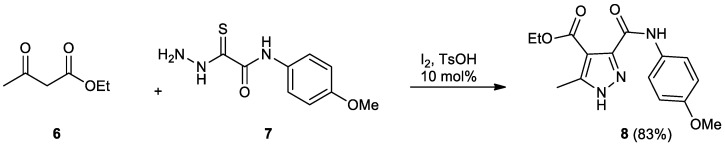
Synthesis of pyrazole derivative **8**.

Iodine was used as a halogenated agent that enhances the cyclization process. Starting with ethyl acetoacetate (**6**) and oxamic acid thiohydrazide **7** as model substrates (Scheme 3), using an equimolar amount of I_2_ in the presence of 10 mol% of TsOH as an additive, afforded pyrazole derivative 8 in 83% yield within 48 h [41]. Different trials using other halogenated agents, such as Br_2_, NCS, or NBS, were also carried out. Iodine was proven as the proper one that gave high yields. 

**Scheme 4:**
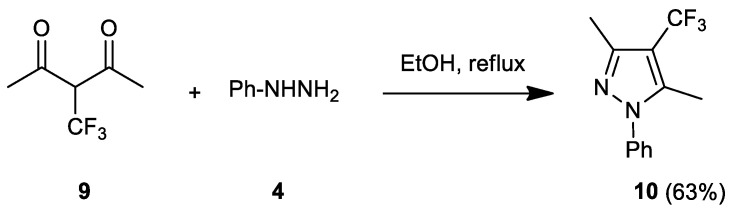
Synthesis of the 1,3,4,5-tetrasubstituted pyrazole **10**.

Ohtsuka et al. [42] prepared 1,3,4,5-tetrasubstituted pyrazole **10** in 63% yield by the condensation of phenyl hydrazine (**4**) with the 2-(trifluoromethyl)-1,3-diketone (**9**) in refluxing ethanol (Scheme 4) [42]. 

**Scheme 5:**
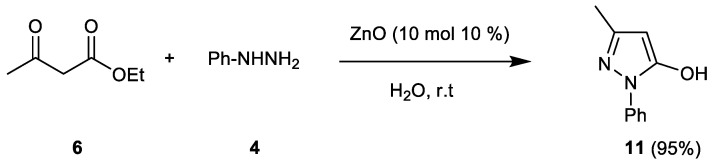
Synthesis of 3-methyl-1-phenyl-1*H*-pyrazol-5-ol (**11**).

Girish et al. [43] showed an efficient nano-ZnO procedure that catalyzed the preparation of 3-methyl-1-phenyl-1*H*-pyrazol-5-ol (**11**) in excellent yield (95%) during the condensation reaction between ethyl acetoacetate and phenylhydrazine (Scheme 5) [43]. Table 1 summarizes the experimental trials used and the optimal conditions of the chosen catalyst and its concentrations. 

**Scheme 6:**
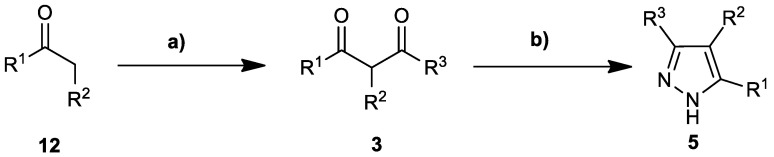
Synthesis of trisubstituted pyrazoles **5**. Reagents and conditions; (a) i—2.1 eq LiHMDS, Toluene/THF, 0 °C, 1 min, ii—1 eq R^3^COCl, r.t, 1 min; (b) i—34 eq NH_2_NH_2_·_2_O, ii—EtOH/THF/Toluene/AcOH (10:7:5:5), reflux 5 min.

In 2006, Heller and Natarajan synthesized pyrazoles **5** from the reaction between hydrazine and 1,3-diketones (Scheme 6). The diketo compounds **3** were successfully prepared in good yields by lithiation, using lithium bis(trimethylsilyl)amide (LiHMDS), followed by subsequent addition of the acid chlorides (Scheme 6) [38].

**Scheme 7:**
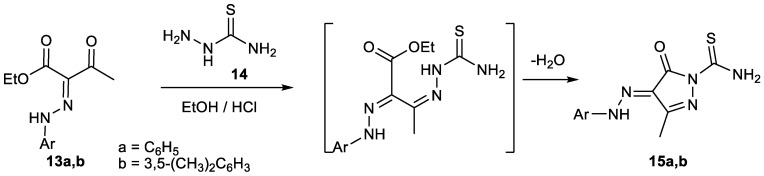
Synthesis of pyrazoles **15a**,**b**.

3-Methyl-5-oxo-4-(2-arylhydrazono)-4,5-dihydro-1*H*-pyrazole-1-carbothioamides **15a**,**b** obtained from the reaction of ethyl 3-oxo-2-(2-arylhydrazono)butanoates **13a**,**b** with thiosemicarbazide (**14**) (Scheme 7) [44]. Reaction proceeds via condensed products are shown in Scheme 7.

#### 2.1.2. With α,β-Unsaturated Ketones

**Scheme 8:**
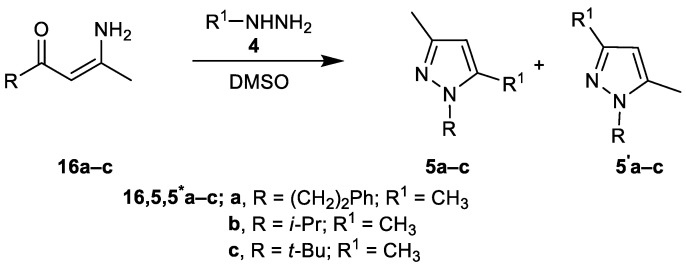
Synthesis of regioselective pyrazoles **5a**–**c** and **5′a**–**c**.

The regioselectivity of the reaction of various *β*-aminoenones on different monoalkyl, acetyl-, methoxycarbonylhydrazine, and semicarbazide was studied by Alberola et al. [45]. They found that the smallest bulky group, when attached at the *β*-position of the enone, obtained high regioselectivity from the reaction of *β*-aminoenones **16a**–**c**, which possessed the least bulky substituent (CH_3_) in the *β*-position with alkyl hydrazines **4**, in DMSO. Subsequently, pyrazoles **5a**–**c** and **5′a**–**c** were obtained with high regioselectivity (Scheme 8) [45]. When different *β*-aminoenones **16a**–**c** with bulkier *β*-substituents were used, the reactivity towards product formation decreased, but more important than this decrease in reactivity was the drop in regioselectivity. This phenomenon was greater when R^1^ and the alkyl hydrazine were bulkier [45]. Compounds **5a**–**c** were formed in yield percentages from 78–97% compared with their regioisomers **5′a**–**c** [45].

**Scheme 9:**
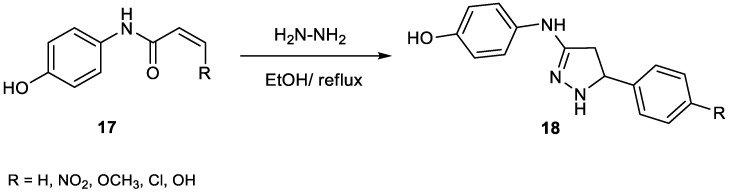
Synthesis of 4-(5-substituted aryl-4,5-dihydropyrazole-3-yl-amino)phenols **18**.

Sahu et al. [46] prepared a group of 4-(5-substituted aryl-4,5-dihydropyrazole-3-yl-amino)phenols **18** (Scheme 9) from the reaction of *N*-(4-hydroxyphenyl)-3-phenylacrylamides **17** with hydrazine hydrate [46]. 

**Scheme 10:**
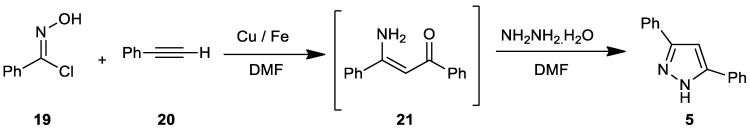
Synthesis of 3,5-diphenyl-1*H*-pyrazole **5**.

Kovacs et al. [47] developed a technique for preparing 3,5-disubstituted pyrazole **5** via Cu/Fe catalyzed coupling between phenylacetylene (**20**) and an oxime **19** in DMF as a solvent provided the *β*-aminoenone **21**. In the one-pot procedure, the valuable *β*-aminoenone was transformed into **5** with the addition of hydrazine hydrate (Scheme 10) [47].

**Scheme 11:**
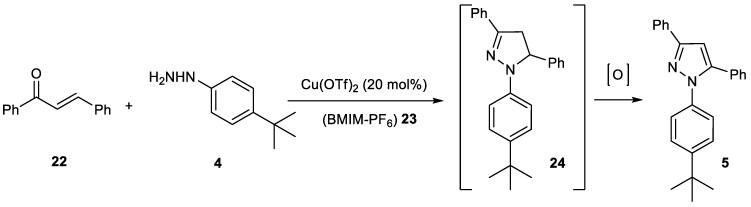
Synthesis of 1,3,5-trisubstituted pyrazole **5**.

Rao et al. [48] described a method to prepare pyrazole derivative **5** via condensation of a chalcone **22** with *p*-((*t*-butyl)phenyl)hydrazine **4** in the presence of copper triflate and 1-butyl-3-methylimidazolium hexafluorophosphate [BMIM-PF_6_] **23** as a catalyst. The reaction proceeded via the formation of compound **24** (Scheme 11) [48]. Further optimization of the reaction conditions was carried out by changing solvents, catalysts, and catalyst loading. The use of 20 mol% Cu(OTf)_2_ in **23** gave the desired product **5** in excellent yield (82%). When Cu(OTf)_2_ was replaced with other catalysts, such as *p*-TSA, Sc(OTf)_3_, Ce(OTf)_3_, Zn(OTf)_2_, AgOTf, or Yb(OTf)_3_, a mixture of **24** and **5** was observed. The use of Ce(OTf)_3_ in **23** resulted in a 75% yield of **24** along with 10% of **5**, whereas the use of *p*-TSA in **23** gave 69% of **24**. The obtained data indicate that Cu(OTf)_2_ was involved in the aerobic oxidation of **24** to **5**. It is necessary to mention that **5** was not formed in the absence of Cu(OTf)_2_ in **23** ionic liquids, and only **24** was isolated in 20% yield along with the starting material, and the yield of **24** did not increase with increasing the time up to 2 h [48].

**Scheme 12:**
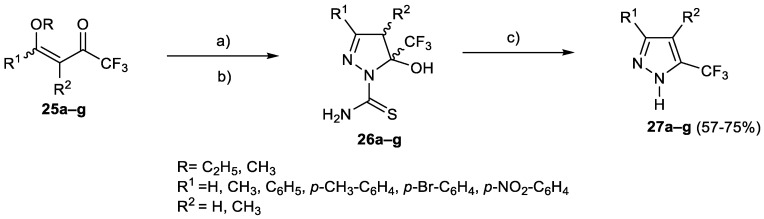
Synthesis of 3-aryl(alkyl)-5-triflfluoromethyl-1*H*-pyrazoles **27a**–**g**. Reagents and conditions; (a) NH_2_NHC(S)NH_2_ (**14**), CH_3_OH; (b) r.t. to 45 °C, 20–24 h; (c) H_2_SO_4_ 96%, reflux, 4 h.

Bonacorso et al. [49] synthesized a series of 3-aryl(alkyl)-5-triflfluoromethyl-1*H*-pyrazoles **27a**–**g** from the reaction of 4-alkoxy-4-aryl(alkyl)-1,1,1-triflfluoro-3-buten-2-ones **25** with thiosemicarbazide (**14**). The reaction gave the corresponding 5-hydroxy-5-triflfluoromethyl-1-pyrazole thiocarboxamides **26**. Subsequently, dehydration and removal of the thiocarboxyamide group with sulfuric acid 96% produced the desired products **27a**–**g** in 57–75% yields (Scheme 12) [49]. It was concluded that the presence of the thiocarboxyamide group on position 1 of the pyrazolines **26** acts as a protective group with an electron-withdrawing effect, hindering the elimination of water and the subsequent aromatization of the five-membered ring. The presence of a trifluoromethyl group on the vinyl ketones **25** and the thiocarboxyamide group on the dinucleophile (thiosemicarbazide) was the determining factor of the regiochemistry of the reaction. Moreover, the presence of α-alkyl- and β-alkyl[aryl]-substituent on the vinyl ketones **25** did not show observable effects on the regiochemistry of the reaction.

**Scheme 13:**
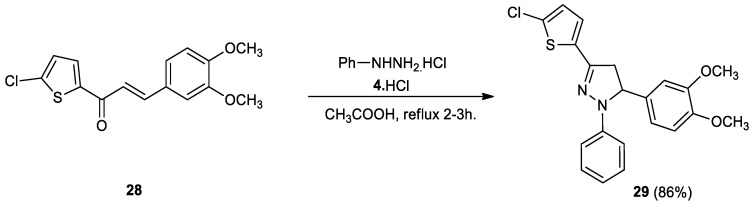
Synthesis of pyrazole derivatives **29**.

Synthesis of pyrazoles substituted by thiophene moiety **29** could be carried during the reaction of chalcone-type compound **28** with phenyl hydrazine hydrochloride **4**-HCl via 3 + 2 annulations (Scheme 13). The obtained thiophene-pyrazole hybrids **29** were screened as antimicrobial and antioxidant agents (Scheme 13) [50].

**Scheme 14:**
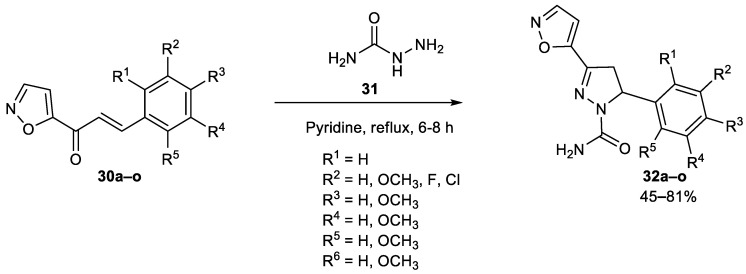
Synthesis of isoxazole appended 1-carboxamido-4,5-dihydro-1*H*-pyrazoles **32a**–**o**.

A series of dihydropyrazole-1-carboxamides **32a**–**o** were obtained by the base-catalyzed condensation of isoxazolyl chalcones **30** with semicarbazide (**31**) (Scheme 14) [51]. The preliminary in vitro antitubercular activity of the synthesized pyrazoles **32a**–**o** was performed by the microplate Alamar Blue assay (MABA) using isoniazid (0.25 μg/mL) as the positive control. 

**Scheme 15:**
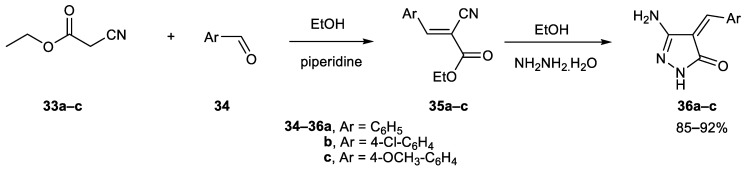
Synthesis of pyrazole derivatives **36a**–**c**.

Similarly, pyrazole derivatives **36a**–**c** were obtained via reaction of *α*,*β*-unsaturated ketones **35,** together with hydrazine, as indicated in Scheme 15. The carboxylated multi-walled carbon nanotubes/dolomite (MWCNTs) successfully grafted the surface of the obtained compounds. Good antibacterial activity toward some pathogenic types of bacteria was found for the synthesized compounds [52].

#### 2.1.3. With Acetylenic Compounds

**Scheme 16:**
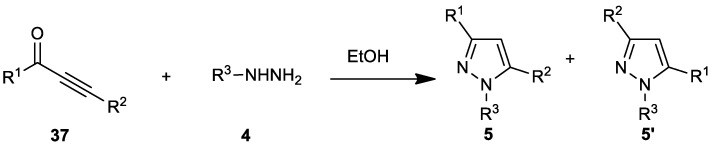
Cyclocondensation reaction of hydrazine derivatives on the acetylenic ketones **37**.

The cyclocondensation reaction of acetylenic ketones **37** with hydrazine derivatives **4** yielded nearly equal yields percentages of the two regioisomers **5** and **5′** (Scheme 16) [53]. 

**Scheme 17:**
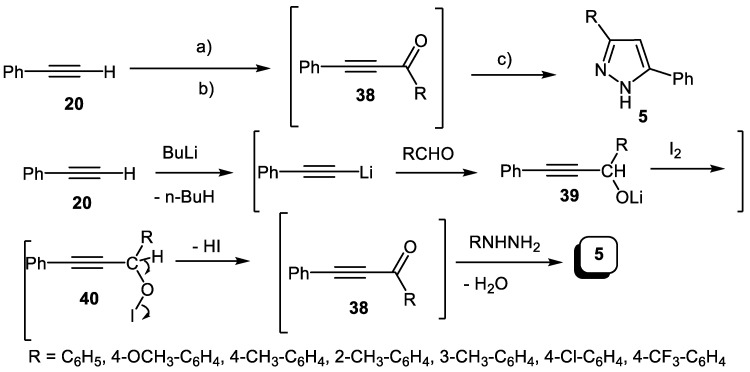
Synthesis of 3,5-disubstituted pyrazole **5**. Reagents and conditions; (a) *n*-BuLi (1.1 eq), THF, 0 °C; (b) ArCHO **34**/ I_2_(1.05 eq); (c) RNHNH_2_ **4** (2.0 eq).

Harigae et al. [54] reported on the synthesis of 3,5-disubstituted pyrazole **5** with high regioselectivity in a one pot procedure via the reaction of phenylacetylene (**20**) with aromatic aldehydes **34**, molecular iodine, and hydrazines **4** (Scheme 17) [54]. The mechanism explains the formation of acyl phenylacetylene **38** due to the lithiation process that generates an acetylenic nucleophilic site and attacks the aldehydic carbonyl to form the intermediate **39** (Scheme 17). Subsequently, the formed nucleophilic center would attack to the iodine molecule to form the intermediate **40**, which executes HI to form **38** (Scheme 17) [54]. The formed intermediate **38** with substituted hydrazines would give compounds **5** accompanied by the elimination of H_2_O (Scheme 17).

**Scheme 18:**
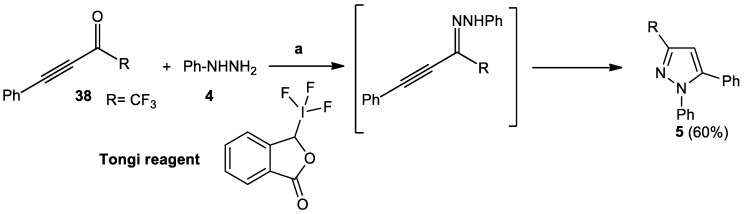
Synthesis of 3-trifluoromethylpyrazoles 5. Reagents and conditions; (a) Togni reagent, CH_3_CN, H_2_O (20:1), r.t., 24 h.

Ji et al. [55] reported on an efficient procedure for synthesizing 3-trifluoromethylpyrazole **5** in 60% yield via trifluoromethylation/cyclization of acetylenic ketones **38** with phenylhydrazine (**4**) using (1-trifluoromethyl-1,2-benziodoxol-3(1*H*)-one) (Togni reagent) (Scheme 18) [55]. 

**Scheme 19:**
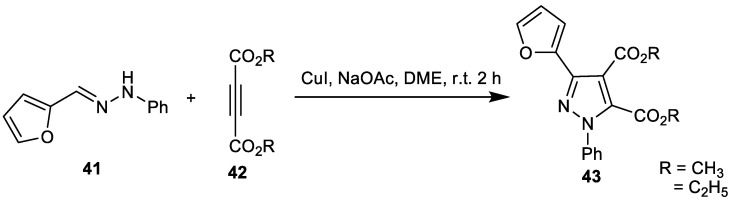
Synthesis of polysubstituted pyrazoles **43** (**41** (0.2 mmol), **42** (0.2 mmol), base (0.2 mmol), catalysts (0.02 mmol) in 2 mL of solvent for 2 h under air).

Ma et al. [56] developed an efficient copper-catalyzed reaction to prepare polysubstituted pyrazoles **43** from phenylhydrazones **41** and dialkyl acetylenedicarboxylates **42** (Scheme 19). Table 2 summarizes the reaction conditions from the molar ratios of the catalyst and base. Moreover, the reaction yields the products in the absence of a catalyst and case of nitrogen atmosphere. The best condition was equal equivalents of the starting substances, base, catalyst, and N_2_ atmosphere [56].

**Scheme 20:**
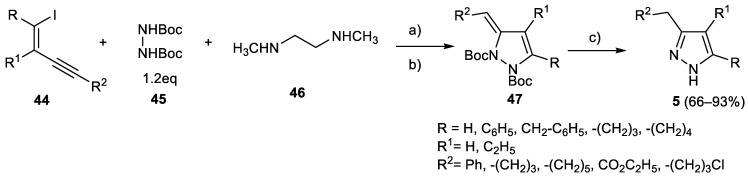
Synthesis of pyrazoles **5**. Reagents and conditions; (a) 5 mol% CuI; (b) 1.5 eq Cs_2_CO_3_, THF, 80 °C, 6–16 h, (c) 10 eq TFA, CH2Cl2, r.t.-2h.

Martín et al. [57] reported a facile method in preparing pyrazoles **5** via Cu-catalyzed domino C-N coupling hydroamination reaction (Scheme 20). The procedure involving the reaction of acetylenes **44** and diamine **45** in the presence of copper (I) iodide and *N^1^*,*N^2^*-dimethylethan-1,2-diamine (**46**) under reflux of THF at 80 °C to give **47** and then pyrazoles **5** were formed in 66–93% yields (Scheme 20) [57].

**Scheme 21:**
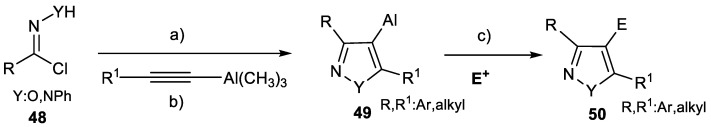
Synthesis of trisubstituted pyrazoles **50**. Reagents and conditions; (a) Al(CH_3_)_3_, toluene, r.t. = 5 min; (b) 1 eq, 50 °C, 1 h; (c) E+ = (EX): D (CH_3_OD, Cl (NCS), I (NIS), CONH_2_ (Cl_3_C-NCO).

In 2011, Jackowski et al. showed heterocycles **50** could be obtained by a simple metalation cyclization process (Scheme 21). 

The carbon–aluminum bond can react further with several electrophiles without the need for transmetalation, providing direct access to trisubstituted pyrazoles **50** (Scheme 21) [58].

#### 2.1.4. With π-Deficient Compounds

**Scheme 22:**
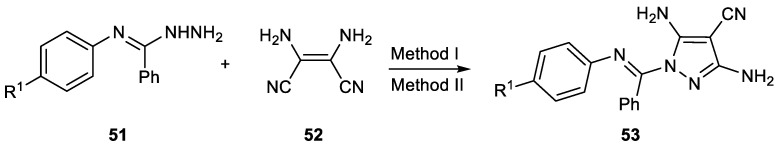
MW assisted synthesis of pyrazoles **53**. Reagents and conditions. Method (I) EtOH/Et3N, reflux 10–16 h (10–25%). Method (II) MW (75–87%).

Aly et al. reported that *N*-arylbenzamidrazones **51** reacted with diaminomaleonitrile (**52**) in EtOH/Et_3_N (Method I) to give substituted pyrazoles **53** (Scheme 22). When microwave irradiation assisted the former reaction for a few min, the corresponding compounds **53** were obtained in good yields (75–87%, Method II, Scheme 22) [59].

**Scheme 23:**
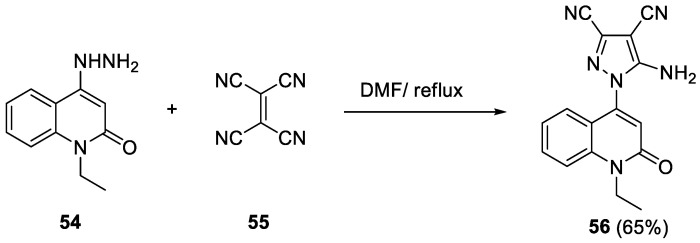
Synthesis of 4-pyrazoloquinolin-2-one **56**.

In addition, Aly et al. prepared 5-amino-1-(1-ethyl-2-oxo-1,2-dihydroquinolin-4-yl)-1*H*-pyrazole-3,4-dicarbonitrile (**56**) from the reaction 2-quinolonyl hydrazine **54** with 1,1,2,2-ethenetetracarbonitrile (**55**) (Scheme 23). Compound **56** was evaluated as good antiproliferative EGFR-TK inhibitors against many tumor cell lines (Scheme 23) [60].

**Scheme 24:**
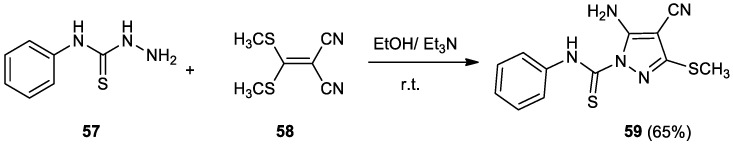
Synthesis of pyrazole **59**.

Gentle heating at 50 °C of equimolar solutions of *N*-phenylhydrazinecarbothioamide (**57**) and 2-bis(methylthio)methylene)malononitrile (**58**) in absolute ethanol containing 0.5 mL Et_3_N for 3 h gave compound **59** in 65 % yield (Scheme 24) [61].

**Scheme 25:**
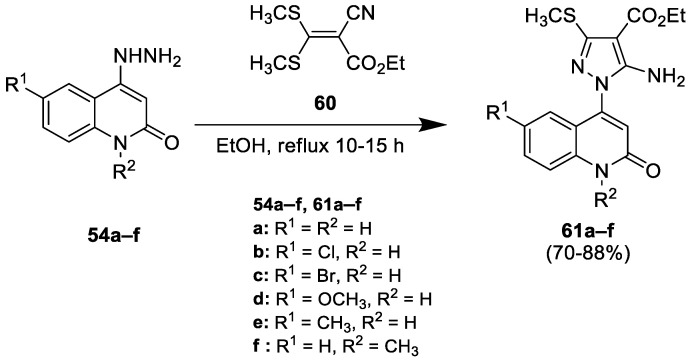
Pyrazoloquinolinones 61a–f with antioxidant and anti-apoptotic activity, and caspase-3 inhibition.

Aly and his co-workers also investigated the antioxidant activity, anti-apoptotic activity, and caspase-3 inhibition of pyrazoloquinolinones **61a**–**f** as described in Scheme 25. Formation of **61a**–**f** was established via the reaction of 2-quinoloyl-4-hydrazines **54a**–**f** with ethyl 2-cyano-3,3-bis(methylthio)acrylate (**60**) (Scheme 25). Compound **61c** was the most potent against inflammation, whereas **61d** showed the most active caspase-3 [61].

**Scheme 26:**
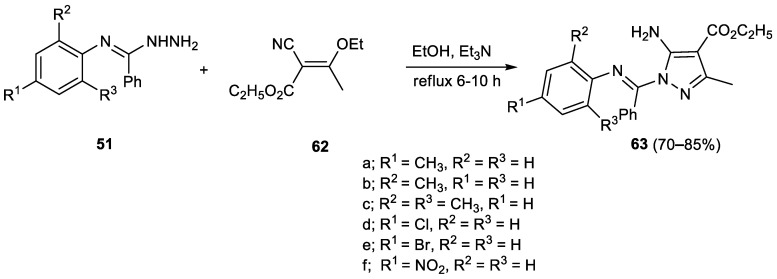
Synthesis of pyrazoles **63**.

In 2017, Aly et al. showed that amidrazones **51** reacted with ethyl 2-cyano-3-ethoxybut-2-enoate (**62**) in refluxing absolute EtOH containing triethylamine (Et_3_N), compounds **63** (70–85%) were obtained, after chromatographic purification and recrystallization (Scheme 26) [62].

**Scheme 27:**
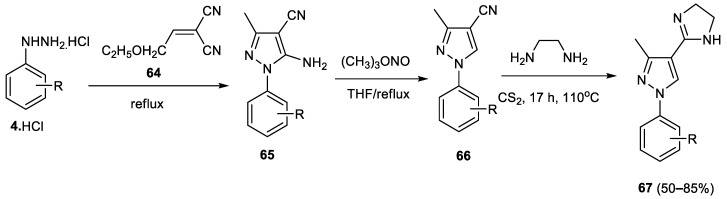
Synthesis of pyrazoles **65**–**67**.

1-Aryl-4-(4,5-dihydro-1*H*-imidazol-2-yl)-3-methyl-1*H*-pyrazoles **67** were obtained in three steps as outlined in Scheme 27.

Compounds **67**, which were obtained in 50–85% yields (Scheme 27), showed good antithrombotic activity in a murine model of arterial thrombosis [63].

**Scheme 28:**
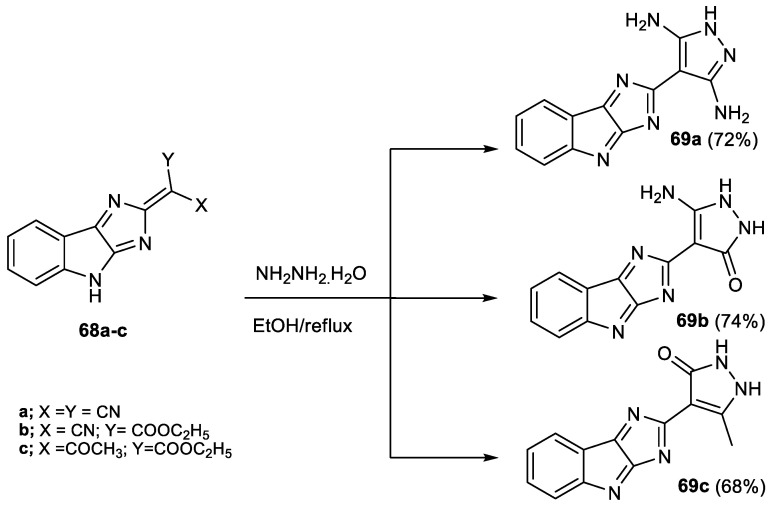
Synthesis of pyrazoles **69a**–**c**.

Synthesis of pyrazole derivatives **69a**–**c** bearing imidazo[4,5-*b*]indole moiety was achieved by the reaction of ylidenes **68a**–**c** with hydrazine hydrate (Scheme 28). The obtained products were successfully examined for their antibacterial activities against four bacterial strains (*Bacillus subtilis*, *Staphylococcus aureus*, *Escherichia coli*, and *Pseudomonas aeruginosa*) and antifungal activities against two fungi (*Aspergillus flavus* and *Candida albicans*) [64].

**Scheme 29:**
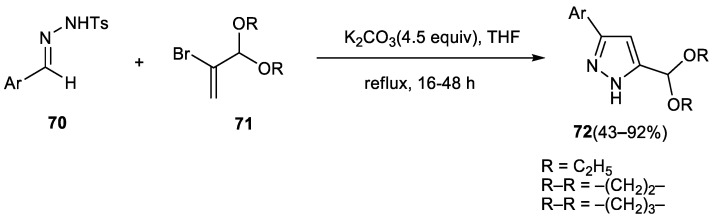
Synthesis of 3,5-disubstituted pyrazoles **72**.

*N*-Tosyl hydrazones **70** with unactivated bromovinyl acetals **71** via 1,3-dipolar cycloaddition reaction to give 3,5-disubstituted **72** pyrazoles was obtained in yields of up to 92% (Scheme 29) [65].

#### 2.1.5. Via Vilsmeier–Haack Reaction

**Scheme 30:**
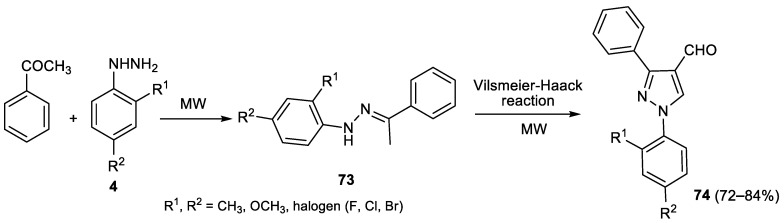
MW assisted the Vilsmeier–Haack reagent in the synthesis of pyrazoles **74**.

In 2014, Selvam et al. [66] reported that when acetophenone, substituted phenyl hydrazine (**4**) in DMF were exposed to MW at 200 W intermittently at 10 s intervals, reaction provided 1-substituted phenyl-2-(1-phenyl-ethylidene)hydrazines 73. When compound 73 was added portion-wise with Vilsmeier–Haack reagent (POCl_3_–DMF/SiO_2_), and the powder is then irradiated in a microwave oven at 400 W intermittently at 30 s intervals, pyrazoles 74 were obtained in moderate to good yields (Scheme 30) [66].

**Scheme 31:**
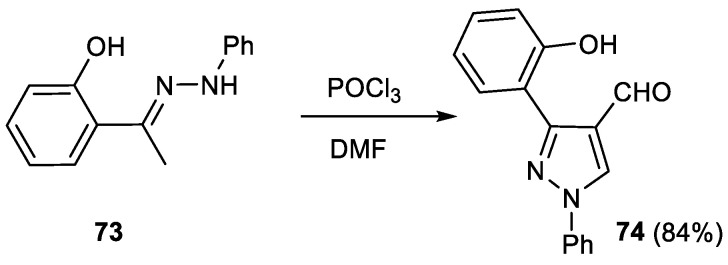
Synthesis of pyrazole-4-carbaldehyde **74**.

Lokhande et al. prepared 3-(2-hydroxyphenyl)-1-phenyl-1*H*-pyrazole-4-carbaldehyde (**74**) using the Vilsmeier–Haack reaction. The condensation process of a hydrazone **73** in POCl_3_/DMF as a solvent gave the 4-formyl pyrazole derivative **74** (Scheme 31) [67].

**Scheme 32:**
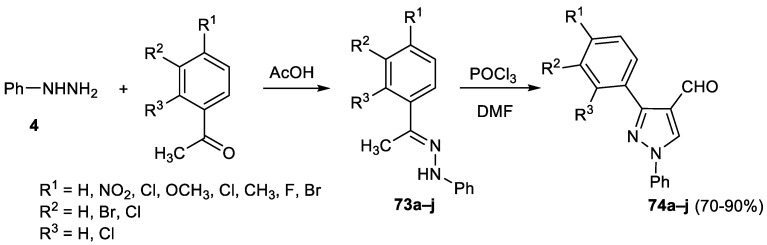
Synthesis of pyrazole-4-carbaldehyde derivatives **74**.

A series of pyrazole derivatives **74a**–**j** has been obtained from arylhydrazones **73a**–**j** via a Vilsmeier–Haack reaction. Among them, a *p*-nitrophenyl moiety connected to a pyrazole scaffold exerted the highest anti-inflammatory activity, which is superior to the standard diclofenac sodium (Scheme 32) [68].

**Scheme 33:**
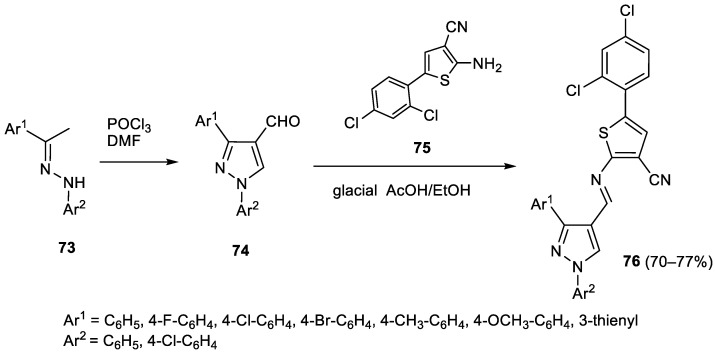
Pyrazole-clubbed thiophene derivatives **76**.

A series of pyrazole derived by thiophene derivatives **76** was achieved. As 1,3-disubstituted-1*H*-pyrazole-4-carbaldehydes **74** were synthesized by Vilsmeier–Haack reaction of hydrazones **73** (Scheme 33). Subsequently, a reaction of **74** with 2-amino-5-(2,4-dichlorophenyl)thiophene-3-carbonitrile (**75**) in the presence of a catalytic amount of glacial acetic acid in ethanol provided **76** in good yields (Scheme 33) [69]. The obtained products were then evaluated for their antibacterial, anti-inflammatory, and antitubercular studies.

**Scheme 34:**
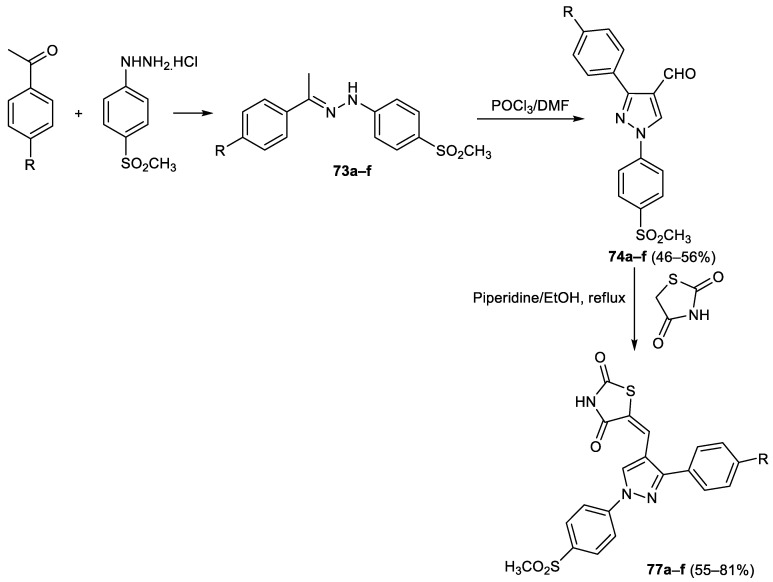
Synthesis of pyrazole sulfonate derivatives **77a**–**f**.

Pyrazole-4-carbaldehyde sulfonate derivatives **74a**–**f** were synthesized via Vilsmeier–Haack reaction hydrazone of sulfonic acids **73a**–**f** with acetophenones. Treatment of compounds **74a**–**f** with thiazolidine-2,4-dione gave the corresponding condensed products **77a**–**f** (Scheme 34) [70].

Compounds **77a**–**f** were evaluated for their COX inhibition, AI activity, ulcerogenic liability, and anti-diabetic activity. The target compounds were assessed in vitro against α-glucosidase and β-glucosidase, in vivo hypoglycemic activity in addition to PPARγ activation study. Two derivatives gave higher COX-2 S.I. (8.69–9.26) than the COX-2 selective drug celecoxib (COX-2 S.I. = 8.60) and showed the highest AI activities and the lowest ulcerogenic than other derivatives. Moreover, these derivatives showed higher inhibitory activities against α- and *β*-glucosidase (% inhibitory activity = 62.15 and 55.30 for α-glucosidase and 57.42 and 60.07 for *β*-glucosidase) than reference compounds (acarbose with % inhibitory activity = 49.50 for α-glucosidase and *D*-saccharic acid 1,4-lactone monohydrate with % inhibitory activity = 53.42 for *β*-glucosidase) and also showed good PPAR-*γ* activation and good hypoglycemic effect in comparison to pioglitazone and rosiglitazone.

**Scheme 35:**
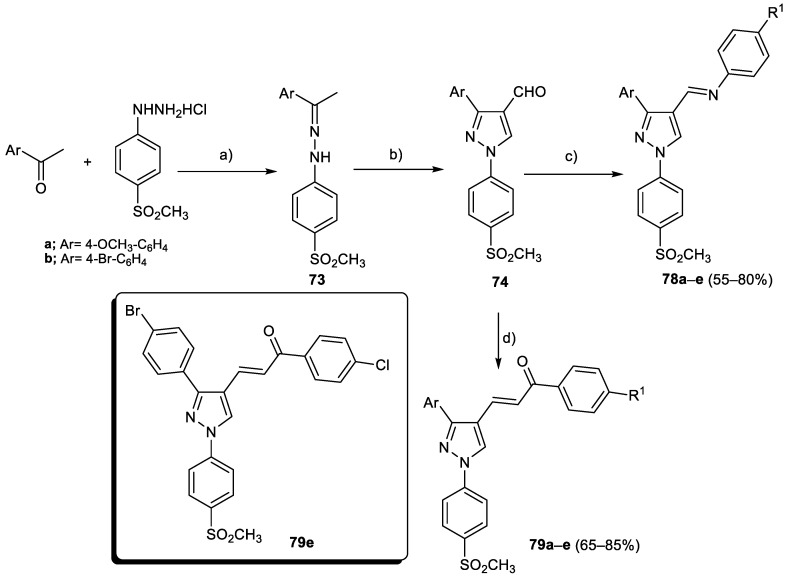
Synthesis of pyrazole derivatives **78a–e** and **79a–e**. Reagents and conditions; (a) glacial AcOH/EtOH, reflux; (b) POCl3/DMF; (c) R1-Ph-NH2, glacial AcOH/EtOH, Reflux; (d) R_2_-Ph-CO-CH_3_, glacial AcOH/EtOH, reflux.

Similarly, two sets of trisubstituted pyrazole derivatives **78a**–**e** and **79a**–**e** were synthesized by the steps shown in Scheme 35. The obtained products were evaluated for their anti-inflammatory effects, cyclooxygenase (COX) inhibitory activity, and ulcerogenic liability (Scheme 35) [71]. Some derivatives of compounds **78a**–**e** and **79a**–**e** showed considerable edema inhibition percentage range compared with celecoxib (13–93% and 58–93%, respectively) at different time intervals. Compound **79e** showed the best screening results if compared with celecoxib (inhibition % = 93.62 and 93.51% at 5 h, COX-1/COX-2 selectivity index SI = 215.44 and 308.16, and ulcer index = 7.25 and 8, respectively).

### 2.2. Pyrazoles from Diazo Compounds

**Scheme 36:**
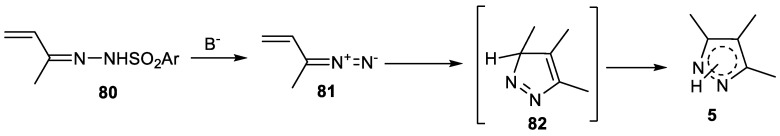
Formation of pyrazoles **5** via intramolecular cycloaddition of vinyldiazonium salt **81**.

Diazoalkanes served as starting materials for the classical Pechmann reaction. Aggarwal et al. reported a one-pot approach proposed using diazo compounds **81** generated in situ from tosyl hydrazone salts **80** (Scheme 36). Direct 1,3-cycloaddition of diazo compounds **81** would afford the pyrazole **5** after an aromatization of the cycloadduct intermediate **82** (Scheme 36) [72].

**Scheme 37:**
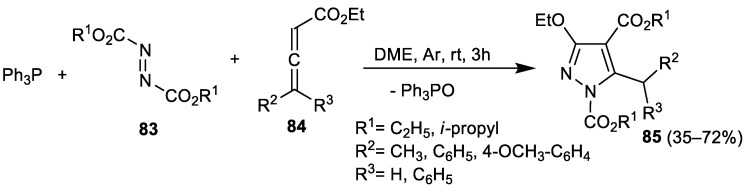
One-pot synthesis of pyrazoles **85**.

1,3,4,5-Tetrasubstituted pyrazoles **85** were synthesized in moderate to good yields through the one-pot reaction of the Huisgen zwitterion from triphenylphosphine in dimethoxyethane (DME) and dialkyl azodicarboxylates **83** with 3-substituted allenoates **84** (Scheme 37) [73].

**Scheme 38:**
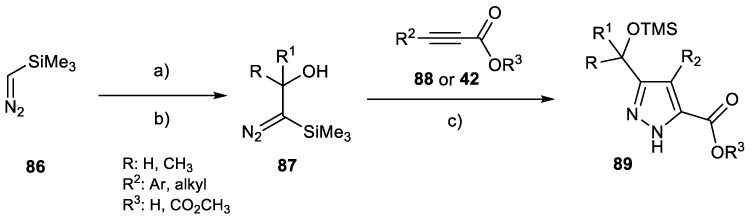
Synthesis of pyrazole derivatives **89**. Reagents and conditions; (a) 1.2 eq. *n*-BuLi/THF, −78 °C, 20 min; (b) 1–1.2 eq.MgBr_2_,10 min, 2.1 eq.RCOR’,1.5 h; (c) THF, reflux, 1 d.

In 2006, Hari et al. [74] synthesized di- and trisubstituted pyrazoles **89** by the reaction of (diazomethyl)trimethylsilane (**86**) with MgBr_2_ and ketones to give **87**, which subsequently reacted with ethyl propiolate (**88**) or dimethyl acetylenedicarboxylate (**42**) under reflux of THF to give pyrazoles **89** (Scheme 38) [74].

**Scheme 39:**
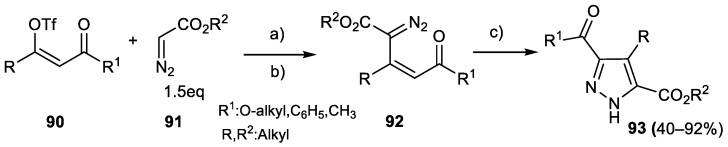
Synthesis of pyrazole derivatives **93**. Reagents and conditions; (a) 5 mol% Pd(PPh_3_)_4_; (b) 2 eq. NMM. DMF, r.t., 3–6 h; (c) 60 °C, 12–18 h.

Cross-coupling/electrocyclization reaction of substituted acyclic and cyclic enol triflates **90** with diazoacetates **91** provided the corresponding 3,4,5-trisubstituted pyrazoles **93** [75]. The reaction of **90** with **91** in the presence of *N*-methylmorpholine (NMM, Scheme 39) was established via the formation of intermediate **92** (Scheme 39) [75].

**Scheme 40:**
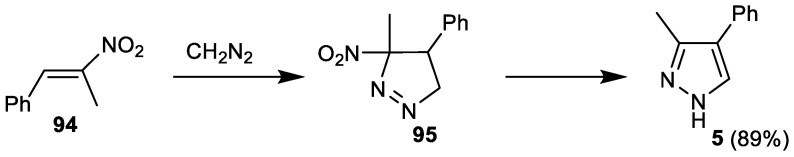
Synthesis of 3-methyl-4-phenyl-1*H*-pyrazole **5**.

Parham et al. [76] developed a facile reaction of (*Z*)-(2-nitroprop-1-en-1-yl)benzene **94** with diazomethane to give pyrazoline **95** that lose the nitro group as oxides of nitrogen on heating, or with acids or bases, to give 3-methyl-4-phenyl-1*H*-pyrazole **5** (Scheme 40) [76].

**Scheme 41:**
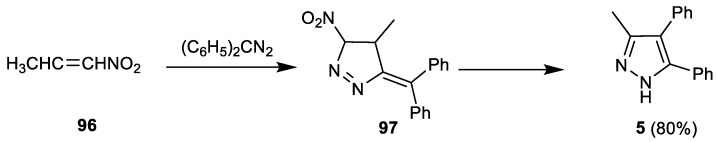
The reaction of diphenyl diazomethane and secondary nitro olefins **96**; synthesis of pyrazole **5**.

The group mentioned above also reported that diphenyl diazomethane and secondary nitroolefin **96** gave 5,5-diphenyl-3-nitro pyrazoline **97**, which rearrange as shown when treated with acids or bases to give 3-methyl-4,5-diphenyl-1*H*-pyrazole **5** (Scheme 41) [77].

**Scheme 42:**
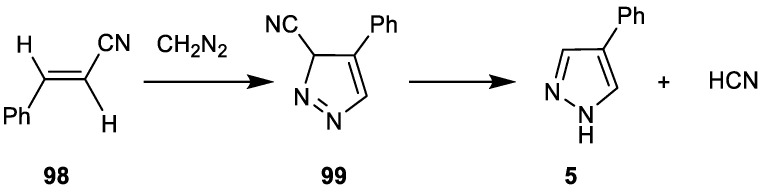
The reaction of cinnamonitrile **98** with diazomethane.

Auwers et al. [78] prepared the pyrazole **5** from the reaction of cinnamonitrile **98** with diazomethane, which gave an unstable pyrazoline **99** that losses hydrogen cyanide and gave **5** (Scheme 42) [78].

**Scheme 43:**
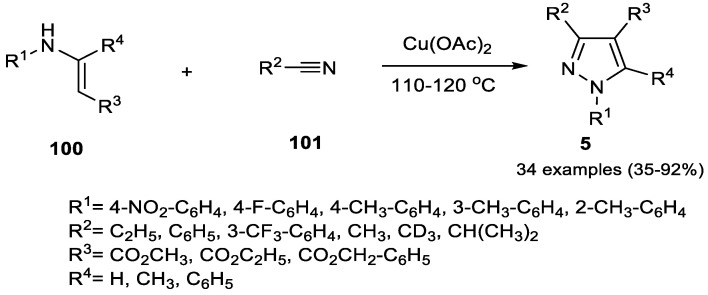
Facile synthesis of tetrasubstituted pyrazoles **5**.

Julia et al. [79] reported on the synthesis of 1,3,4,5-tetrasubstituted pyrazoles **5** (Scheme 43) via the reaction **100** with various substituted nitriles **101** in the presence of cupper acetate at 110–120 °C (Scheme 43) [79].

### 2.3. Heterocyclic Ring Rearrangement

**Scheme 44:**
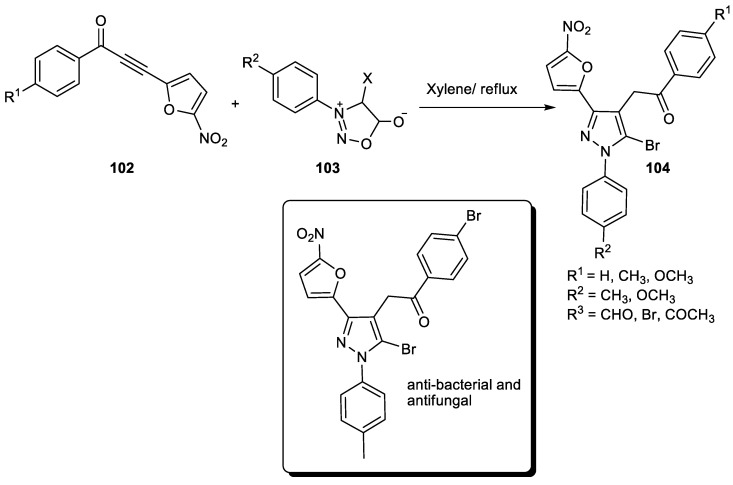
Synthesis of 1,3,4,5-tetrasubstituted pyrazole derivatives **104**.

Rai et al. [80] reported on the synthesis of nitrofuran containing 1,3,4,5-tetrasubstituted pyrazole derivatives **104** (Scheme 44). Compounds **104** were obtained by refluxing 3-(5-nitrofuran-2-yl)-1-phenylprop-2-yn-1-ones **102** with 4-bromo-3-(aryl)-4,5-dihydro-1,2,3-oxadiazol-3-ium-5-olates **103** in xylene. Compound 2-(5-bromo-3-(5-nitrofuran-2-yl)-1-(*p*-tolyl)-1*H*-pyrazol-4-yl)-1-(4-bromophenyl)-ethan-1-one (Scheme 44) showed highest antibacterial and antifungal activity than all other compounds [80].

**Scheme 45:**
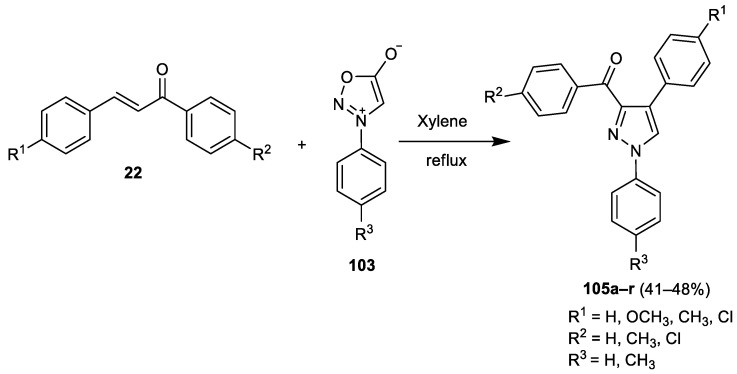
Synthesis of trisubstituted pyrazoles **105**.

Chen et al. conducted the synthesis of a trisubstituted pyrazole **105**, in 41–48% yields, via 1,3-dipolar cycloaddition reaction between arylsydnones **103** and *α*,*β*-unsaturated ketones **22** in dry xylene (Scheme 45) [81].

**Scheme 46:**
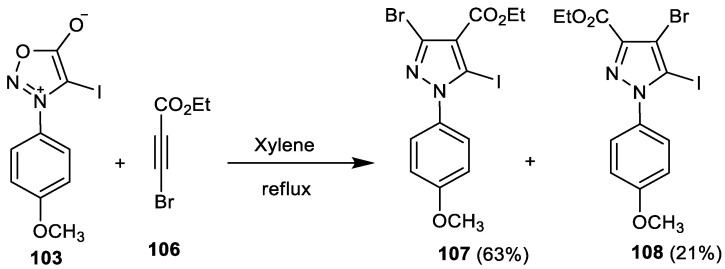
Synthesis of the two regioisomeric **107** and **108**.

Delaunay et al. [82] described the synthesis of the two regioisomeric 1,3,4,5-substituted pyrazoles **107** and **108** by a cycloaddition reaction of a 4-iodo-3-(4-methoxy phenyl)-1,2,3-oxadiazol-3-ium-5-olate **103** with ethyl bromopropiolate (**106**). The separation of pyrazoles **107** and **108** was easily performed by silica gel chromatography (Scheme 46) [82].

**Scheme 47:**
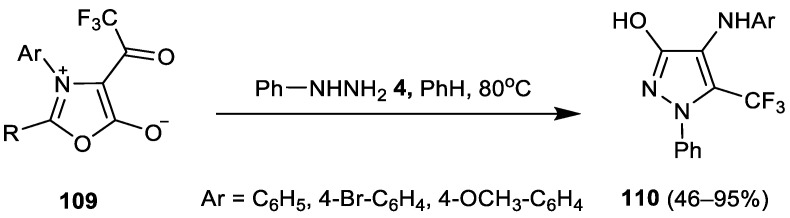
Transformation of oxazoles **109** into pyrazoles **110**.

5-Trifluoromethyl-3-hydroxypyrazoles **110** (Scheme 47) were readily prepared from 4-trifluoroacetyl-1,3-oxazolium-5-olates **109** and phenyl hydrazine (**4**) [83].

**Scheme 48:**
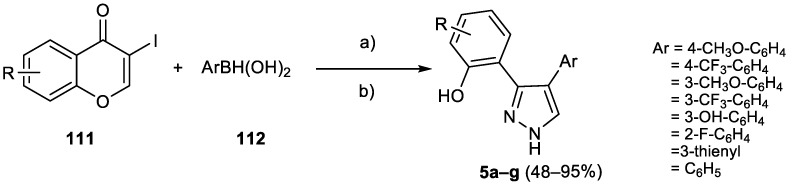
Preparation of 3,4-diarylpyrazoles 5. Reagents and conditions; (a) 2% Pd(PPh_3_)_4_; (b) 2 eq K_2_CO_3_, THF, reflux.

Xie et al. [84] have developed an efficient protocol to prepare 3,4-diarylpyrazoles **5** in 48–95% yields [84]. The strategy involves sequential Suzuki coupling between iodochromones **111** and phenylboronic acids **112** in the presence of Pd(PPh_3_)_4_ and K_2_CO_3_, followed by condensation with hydrazine hydrate (Scheme 48) [84].

**Scheme 49:**
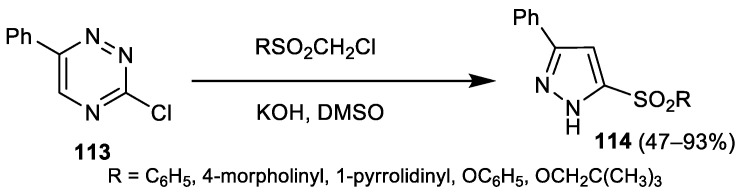
Preparation of pyrazoles **114** from triazines **113**.

Rykowski et al. [85] prepared pyrazoles from triazines by condensation of 3-chloro-6-phenyl-1,2,4-triazines **113** on *α*-chlorosulfonyls in the presence of KOH and DMSO as a solvent to give the corresponding pyrazoles **114** in 47–93% yields (Scheme 49) [85].

**Scheme 50:**
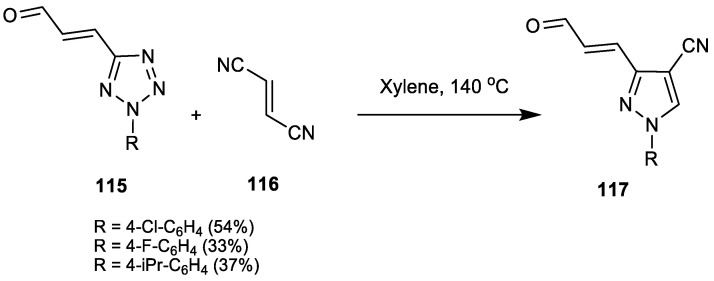
Synthesis of cyanopyrazoles **117**.

In 2000, Simoni et al. [86] reported that tetrazolyl acroleins **115** reacted with fumaronitrile (**116**) in xylene at 140 °C to give the corresponding pyrazoles **117** (Scheme 50) [86].

### 2.4. Multicomponent Synthesis

**Scheme 51:**
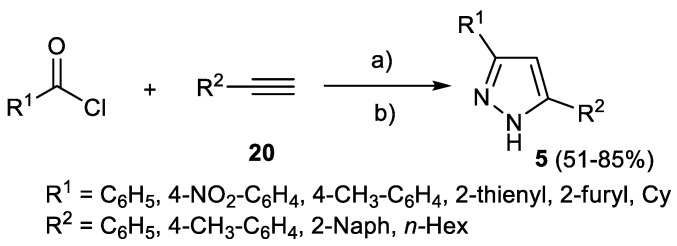
Cyclocondensation of acid chlorides with acetylenes and hydrazine in the presence of PdCl_2_(PPh_3_)_2_/CuI. Reagents and conditions; (a) PdCl_2_(PPh_3_)_2_/CuI, Et_3_N, THF/CH_3_CN, rt 2 h, THF; (b) NH_2_NH_2_/CH_3_CN.

Liu et al. [87] reported on a one-pot, three-component approach consisting of acid chlorides, terminal alkynes, and hydrazine catalyzed by Pd(PPh_3_)_2_Cl_2_/CuI to give 3,5-diaryl-1*H*-pyrazoles **5** in moderate to good yields (Scheme 51). However, the aliphatic alkyne 1-octyne led to its corresponding pyrazole derivative in only 15% yield [87]. A general procedure for the preparation of compounds **5** was described as a mixture of PdCl_2_(PPh_3_)_2_ (0.01 mmol), CuI (0.03 mmol), Et_3_N (2.0 mmol) acid chloride (1.5 mmol), and alkyne **20** (1.0 mmol) in THF (5 mL) was stirred at room temperature for 2 h under N_2_. Then hydrazine (3.0 mmol) in CH_3_CN (2 mL) was added, and the reaction mixture continued to stir for 16 h. The reaction mixture was diluted with water and extracted with dichloromethane. Column chromatography to obtain the pure products **5**.

**Scheme 52:**
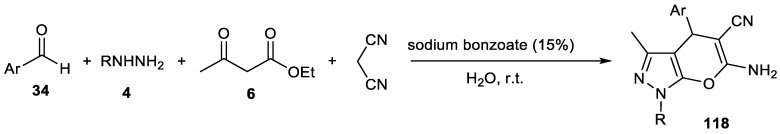
Synthesis 1,4-dihydropyrano[2,3-*c*]pyrazole derivatives **118**.

The four-component reaction of aromatic aldehydes **34**, malononitrile, phenylhydrazine (**4**), and ethyl acetoacetate (**6**) in the presence of sodium benzoate in an aqueous solution (Scheme 52) was reported to give compounds **118** [88]. Sodium benzoate was used as the mild basic catalyst. Table 3 summarizes the trials using different molar % of catalysts and the corresponding yields of products [88].

**Scheme 53:**
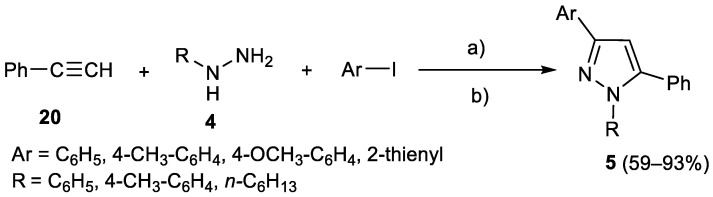
Four component reaction for preparation of pyrazoles **5**. Reagents and conditions; (a) 1 mol, PdCl_2_(PPh)_3_, CO (ambient pressure); (b) THF/H_2_O(1:1), r.t., 24–36 h.

It was reported that the pyrazoles **5** were obtained in 59–93% yields during the reaction of palladium-catalyzed four-component coupling of phenylacetylene (**20**), hydrazine derivatives **4**, aryl iodide, carbon monoxide under ambient pressure, and room temperature for 24 to 36 h (Scheme 53) [89].

**Scheme 54:**
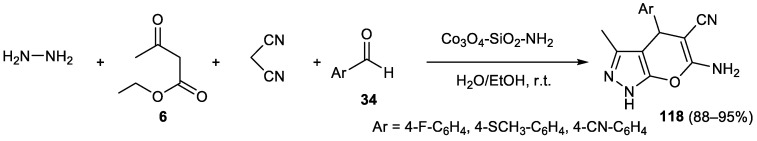
Four component reaction for preparation of pyrazoles **118**.

Pyranopyrazoles **118** were efficiently synthesized in 88–95% yields via the one-pot four-component reactions of ethyl acetoacetate (**6**), hydrazine hydrate, aldehydes **34**, and malononitrile in the presence of Co_3_O_4_-SiO_2_-NH_2_ nanocomposites as a catalyst (Scheme 54) [90].

### 2.5. Eco-Friendly Methods for Pyrazole Synthesis

**Scheme 55:**
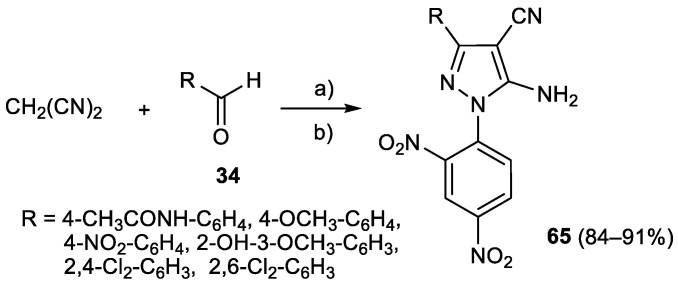
Synthesis of polysubstituted pyrazoles **65** using a deep eutectic solvent. Reagents and conditions; (a) Gly/K_2_CO_3_/H_2_O (4:1:14; W; W), 80 °C, 2 min; (b) 2,4-(NO_2_)_2_-C_6_H_3_NHNH_2_, 80 °C, 18–28 min.

Beyzaei et al. [91] synthesized polysubstituted pyrazoles **65** in 84–91% yields through a two-step, one-pot procedure. In this technique, the reaction of 2,4-dinitrophenylhydrazine, malononitrile, and different aldehydes **34** in deep eutectic solvent (DES) were carried out (Scheme 55) [91].

**Scheme 56:**
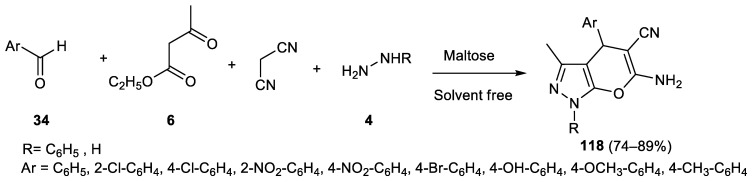
Synthesis of polysubstituted pyrazoles **118**.

Four-component one-pot preparation of 1,4-dihydropyrano[2,3-*c*]pyrazoles **118** using phenylhydrazine **4** or hydrazine monohydrate, acetoacetic ester (**6**), malononitrile, and aldehydes **34** under thermal and solvent-less conditions with maltose as a catalyst was reported by Kangani et al. The reaction efficiently proceeded to produce the respective products **118** in 74–89% yields (Scheme 56) [92].

**Scheme 57:**
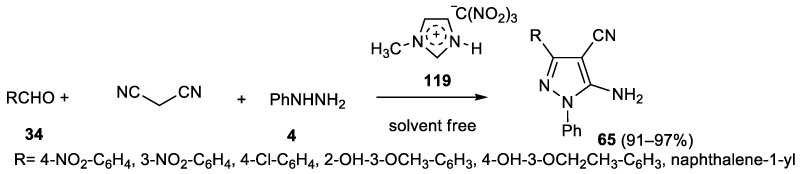
Synthesis of polysubstituted pyrazoles **65**.

Zolfigol et al. described an effective three-component condensation reaction of malononitrile, aryl aldehydes, and phenyl hydrazine (**4**) under solvent-free conditions using 1-methylimidazolium trinitromethanide {[HMIM]C(NO_2_)_3_} (**119**) as a catalyst in the formation of 5-aminopyrazole-4-carbonitriles **65** (Scheme 57) [93].

**Scheme 58:**
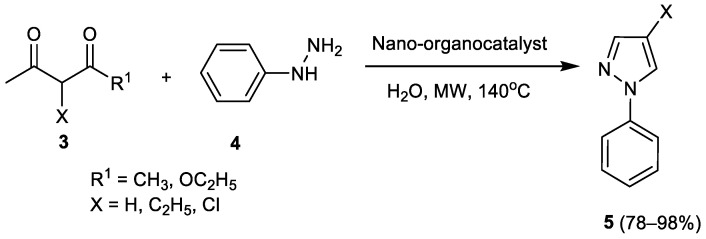
Synthesis of pyrazoles **5**.

Under microwave irradiation, the reaction of 1,3-diketones **3** with phenylhydrazine (**4**) in the presence of organic nanocatalyst in an aqueous medium produced pyrazoles **5** in 78–98% yields (Scheme 58) [94].

**Scheme 59:**
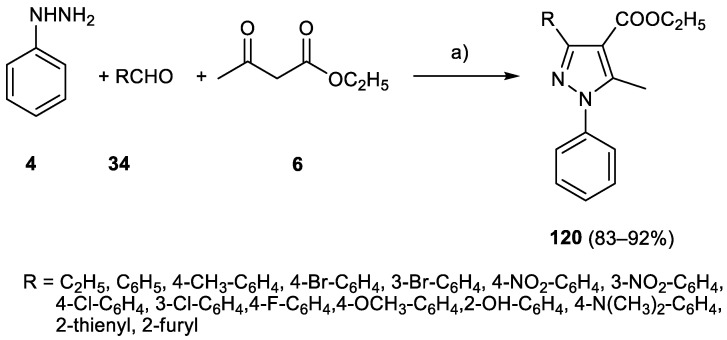
Synthesis of polysubstituted pyrazoles **120**. Reagents and conditions; (a) Sc(OTf)_3_, MW 200 W, 100 °C, 3–6 min.

Facile formation of functionalized pyrazole derivatives **120** under solvent-less conditions was achieved by treating **4** with aldehydes **34** and acetoacetic ester (**6**). This methodology showed the synthetic potential of microwave irradiation and scandium (III) triflate Sc(OTf)_3_ as a catalyst (Scheme 59) [95].

**Scheme 60:**
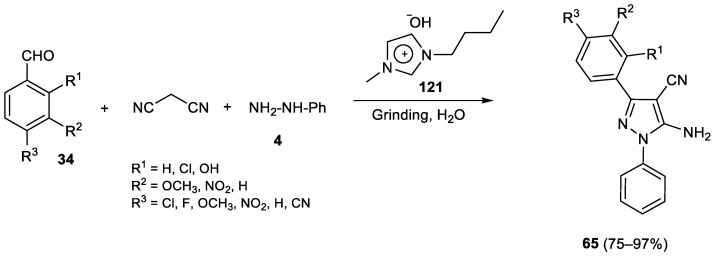
IL mediated formation of highly substituted pyrazoles **65**.

A grinding induced the formation of highly substituted pyrazoles **65** by applying malononitrile, functionalized aldehydes **34**, and phenylhydrazine (**4**). Singh et al. reported this procedure utilizing IL **121** as a catalyst without the formation of any byproducts (Scheme 60). Most importantly, simple handling and attainment of high yield up to 97% are the advantages of this methodology [96].

## 3. Heteroannulated Pyrazoles

### 3.1. Heteroannulation with Six-Membered Heterocycles

#### 3.1.1. Pyranopyrazoles

**Figure 5:**
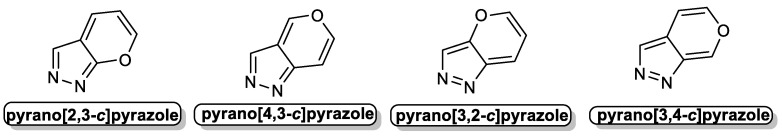
Isomeric structure of pyranopyrazoles.

Pyranopyrazoles consists of four possible isomeric forms naming as pyrano[2,3-*c*]pyrazole, pyrano[4,3-*c*]pyrazole, pyrano[3,2-*c*]pyrazole, pyrano[3,4-*c*]pyrazole and pyrano[2,3-c]pyrazole, are found as the most widely studied (Figure 5).

**Scheme 61:**
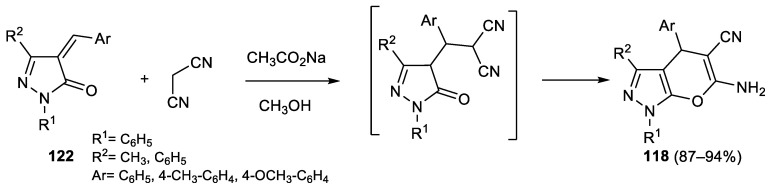
Formation of pyrano[2,3-*c*]pyrazoles **118**.

4-Benzylide-pyrazol-5-one **122** with malononitrile in methanol in the presence of sodium acetate catalyst has been used to obtain pyrano[2,3-*c*]pyrazoles **118** (Scheme 61) [97].

**Scheme 62:**
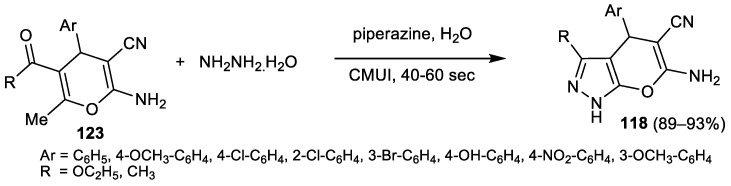
Formation of pyrano[2,3-*c*]pyrazole **118**.

Peng and co-workers reacted 5-alkoxycarbonyl-2-amino-4-aryl-3-cyano-6-methyl-4*H*-pyrans **123** with hydrazine hydrate in the presence of a catalytic quantity of piperazine, and the corresponding pyranopyrazoles **118** were obtained (Scheme 62). The strategy of the synthesis was carried out in three methods, namely (i) heating; (ii) exposure to microwave irradiation; (iii) exposure to a combination of microwave and ultrasound irradiation (CMUI). The procedure was later found to be excellent in yield within a short time (Scheme 62) [98].

**Scheme 63:**
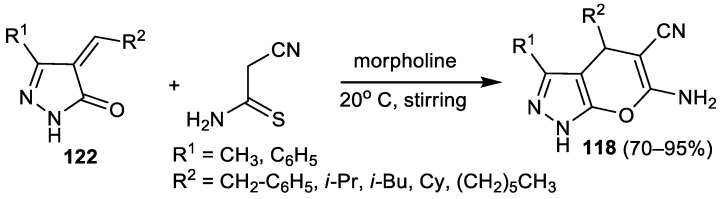
Synthesis of pyranopyrazoles **118**.

Dyachenko and Rusanov allowed stirring benzylidene-pyrazolone **122** with cyanothioacetamide in morpholine containing an ethanolic solution to obtain various pyranopyrazoles **118** (Scheme 63) [99].

**Scheme 64:**
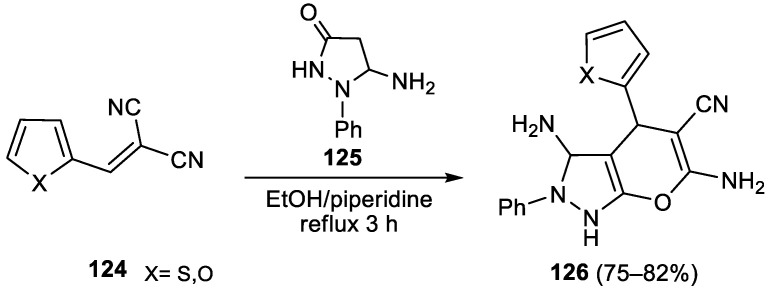
Formation of 3-aminopyrano[2,3-*c*]pyrazoles **126**.

It was reported that the reaction of (2-cyano-3-furan/thiophen-2-yl)acrylonitriles **124** with 3-aminopyrazolin-5-one (**125**) in the presence of the base, which, via Michael addition to afford 3-aminopyrano[2,3-*c*]pyrazoles **126** (Scheme 64) [100].

**Scheme 65:**
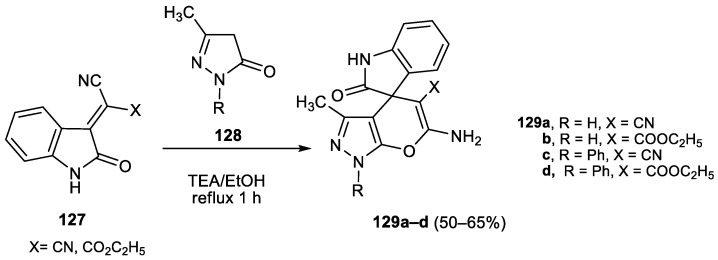
Synthesis of spiropyranylindolones **129a**–**d**.

Hafez and co-workers reacted 2-oxo-3-substituted indole **127** with pyrazolone **128** in boiling ethanol and catalyzed by Et_3_N to prepare spiropyranylindolones **129** (Scheme 65) [101].

**Scheme 66:**
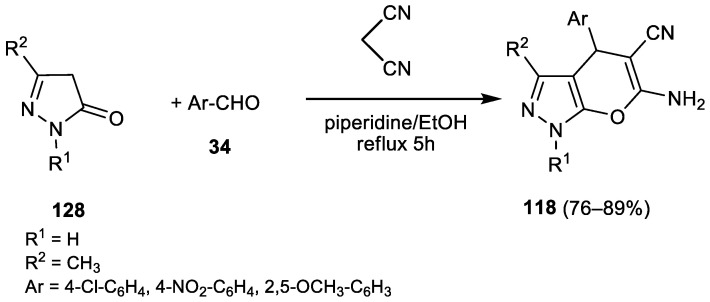
Synthesis of pyranopyrazoles **118**.

Pyranopyrazoles **118** were obtained in good yields by a three-component reaction of aldehydes **34**, malononitrile, and pyrazol-5-one **128**, in refluxing ethanol with piperidine catalyst (Scheme 66) [102].

**Scheme 67:**
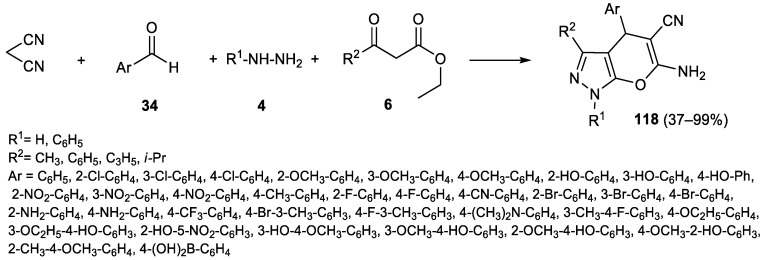
Synthesis of pyranopyrazoles **118**.

Pyrano[2,3-*c*]pyrazoles **118** were prepared by a four-component reaction of aldehydes **34**, malononitrile, β-ketoester (**6**), and hydrazines **4** with/without catalyst. Several carbonyl compounds, β-keto ester, and un/substituted hydrazine were chosen together with various catalysts, solvents, temperatures, and green techniques, which were also applied (Scheme 67) [103].

**Scheme 68:**
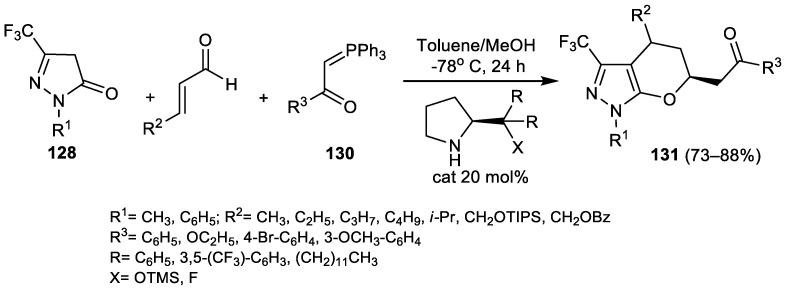
Enantioselective formation of tetrahydropyrano-pyrazoles **131**.

Enders and co-workers prepared the enantioselective tetrahydropyrano-pyrazoles **131** from the reaction of pyrazolone **128**, α,β-unsaturated aldehydes, and Wittig reagent **130** in the presence of secondary amines, such as catalysts (Scheme 68) [104].

**Scheme 69:**
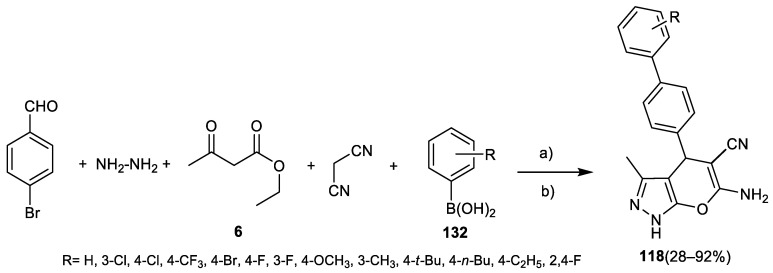
Multicomponent synthesis of pyrano-pyrazoles **118**. Reagents and condition: (a) Pd/C, DMF, 1 mol% Pd; (b) 50% DMF, KF·2H_2_O, 80 °C.

Lu and co-workers reported on a one-pot synthesis of pyranopyrazoles **118** via Suzuki coupling between 4-bromobenzaldehyde and aryl boronic acid **132** together with KF·2H_2_O as a dehalogenating agent in the presence of Pd/C at 80 °C. Firstly, 4-bromobenzaldehyde and aryl boronic acid was added to form substituted biphenyl aldehydes; subsequently, other reagents were added and allowed to react for 5–6 h (Scheme 69) [105].

**Scheme 70:**
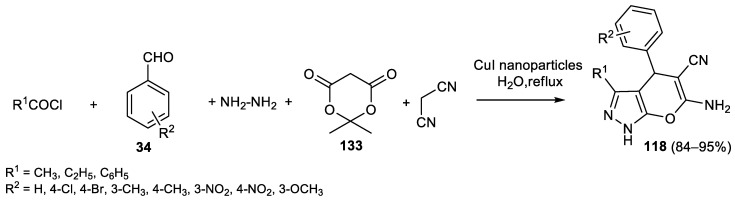
Multicomponent synthesis of pyrano-pyrazoles **118**.

Another five components, synthesis of pyranopyrazoles **118,** involved a mixture of acid chlorides, Meldrum’s acid (**133**), aromatic aldehydes **34**, hydrazine hydrate, and malononitrile in the presence of CuI nanoparticles (Scheme 70) [106].

#### 3.1.2. Pyrazolopyrimidine

**Figure 6:**
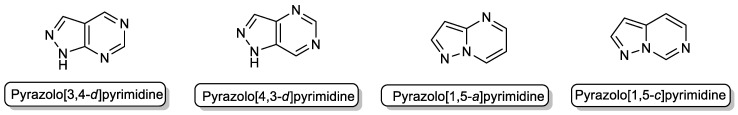
Isomeric forms of pyrazolopyrimidines.

Pyrazolopyrimidines are considered the structural analogs of the biogenic purine class. Pyrazolopyrimidines are of interest as potential bioactive molecules. Pyrazolopyrimidines have four known structures, as illustrated in Figure 6.

**Figure 7:**
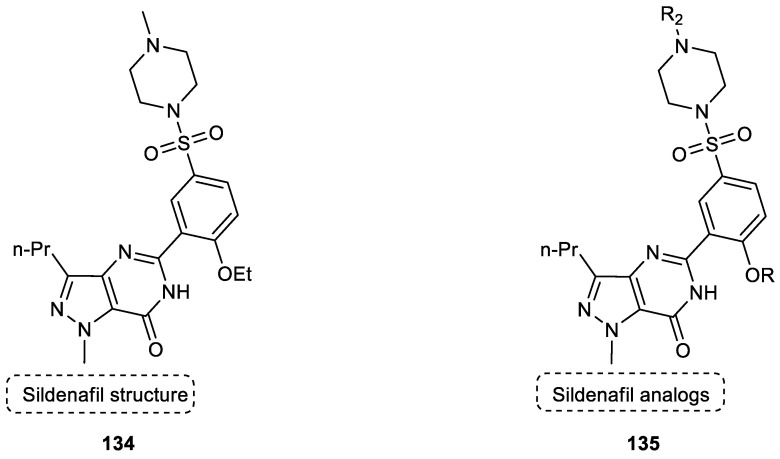
Chemical structure of Sildenafil **134** and Sildenafil analog **135**.

One of the essential pharmacological applications of pyrazolo[4,3-*d*]pyrimidine derivatives is Sildenafil (Viagra^®®^, **134**) and its analogs **135** (Figure 7). Compounds **135** were used as a selective phosphodiesterase 5 (PDE5) to treat male erectile dysfunction as an oral agent. Recently, a series of Sildenafil analogs (R = Me, Et; R2 = Me, Et, -CH_2_CH_2_OH) was prepared, and the in vitro PDE5 inhibitory activities were evaluated; the results revealed improved activity and selectivity [107].

**Scheme 71:**
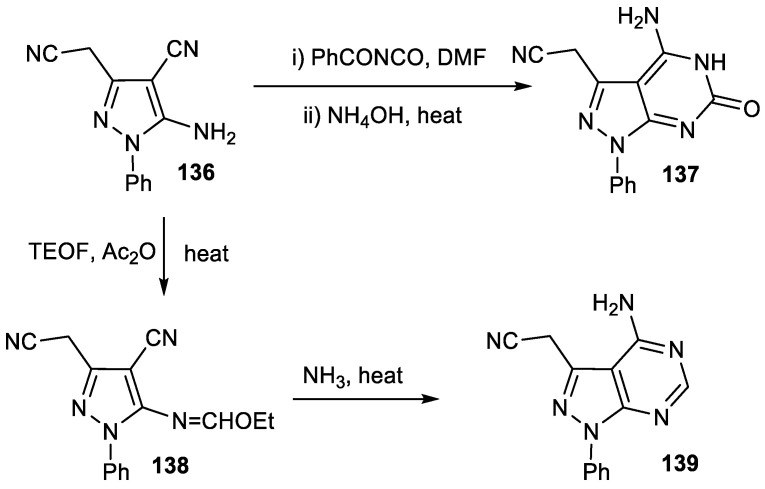
Synthesis of pyrazolopyrimidinone derivatives **137** and **139**.

The reaction of compound **136** with benzoyl isocyanate in the presence of ammonium hydroxide gave 5-amino-1-phenylpyrazolo[3,4-*d*]pyrimidinone derivative (**137**) (Scheme 71) [108]. Treatment of **136** with triethyl orthoformate in acetic anhydride afforded the methanimidate **138**, which on treatment with ammonia gave pyrazolo[3,4-*d*]pyrimidin-4-ylaminederivative **139** (Scheme 71) [109].

**Scheme 72:**
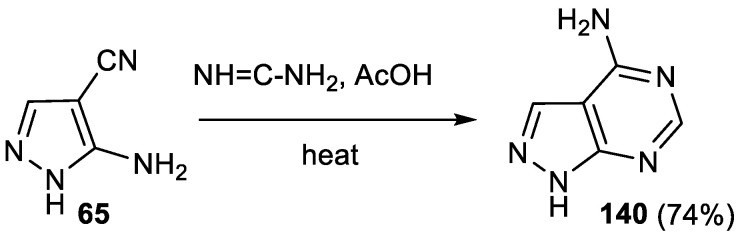
Synthesis of 5-Amino-1*H*-pyrazolo[3,4-*d*]pyrimidine derivative **140**.

5-Amino-1*H*-pyrazolo[3,4-*d*]pyrimidine derivative **140** could be obtained directly by treatment of 5-aminopyrazole-4-carbonitrile (**65**) formamidine in acetic acid (Scheme 72) [110].

**Scheme 73:**
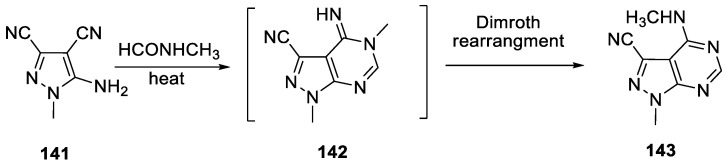
Synthesis of pyrazolo[3,4-*d*]pyrimidine **143**.

The reaction of 5-amino-1-methyl-1*H*-pyrazole-3,4-dicarbonitrile **141** with *N*-methylformamide gave the imine intermediate **142**. The latter intermediate underwent ring opening by a typical Dimroth rearrangement and recyclized to furnish the pyrazolo[3,4-d]pyrimidine **143** carrying a methylamino group at 4-position (Scheme 73) [111].

**Scheme 74:**
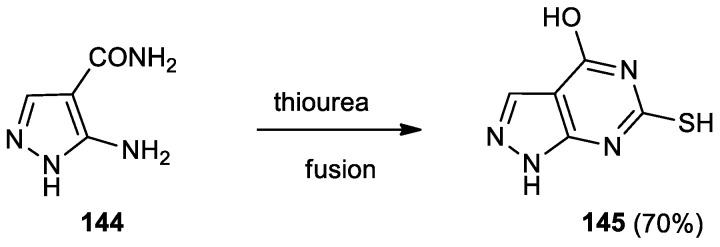
Synthesis of 4-hydroxy-6-mercaptopyrazolo[3,4-*d*]pyrimidine **145**.

Conversion of compound **65** into corresponding carboxylic acid amide derivative **144** was achieved by the hydrolysis of the nitrile group using sulfuric acid. On the fusion of **144** with thiourea, the reaction proceeded to give 4-hydroxy-6-mercaptopyrazolo[3,4-*d*]pyrimidine **145** (Scheme 74) [112].

**Scheme 75:**
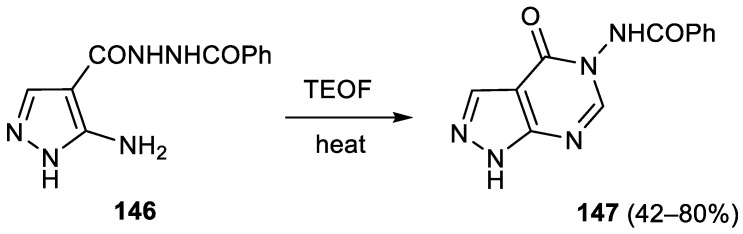
Synthesis of 5-benzamido-1*H*-pyrazolo[3,4-*d*]pyrimidin-4-one **147**.

5-Benzamido-1*H*-pyrazolo[3,4-*d*]pyrimidin-4-one (**147**) was prepared by reacting 5-amino-1*H*-pyrazole-4(*N*-benzoyl)carbohydrazide (**146**) with triethyl orthoformate (TEOF) (Scheme 75) [113].

**Scheme 76:**
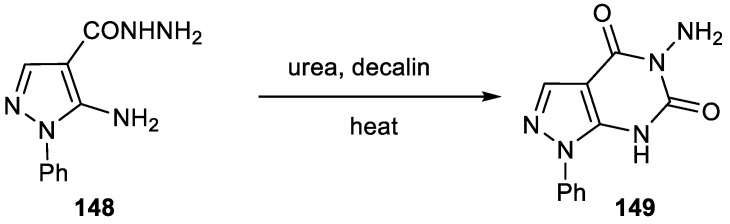
Synthesis of pyrazolo[3,4-*d*]pyrimidine-4,6-dione **149**.

Similarly, reaction of 5-amino-1-phenyl-1*H*-pyrazole-4-carboxylic acid hydrazide (**148**) with urea in decalin gave pyrazolo[3,4-*d*]pyrimidine-4,6-dione derivative **149** (Scheme 76) [114].

**Scheme 77:**
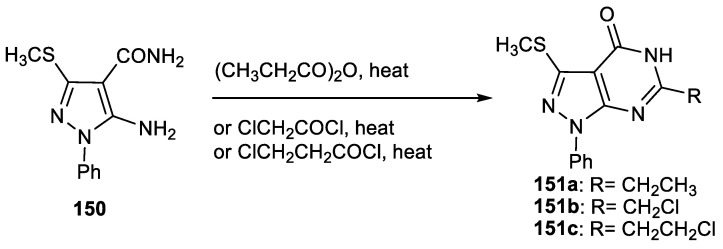
Synthesis of 6-substituted pyrazolo[3,4-*d*]pyrimidin-4(5*H*)-one derivatives **151a**–**c**.

El-Enany et al. [115] reacted 5-amino-3-methylsulphanyl-1-phenyl-1*H*-pyrazole- 4-carboxylic acid amide (**150**) with propionic anhydride, chloroacetyl chloride or 3-chloropropionyl chloride to produce the 6-substituted pyrazolo[3,4-*d*]pyrimidin-4(5*H*)-one derivatives **151a**–**c** in 83–96% yield (Scheme 77) [115].

**Scheme 78:**
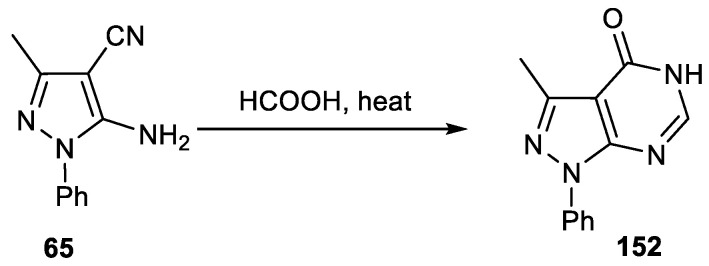
Synthesis of pyrazolo[3,4-*d*]pyrimidin-4(5*H*)-one **152**.

Kandeel et al. [116] synthesized pyrazolo[3,4-*d*]pyrimidin-4(5*H*)-one **152** via the reaction of 5-amino-3-methyl-1*H*-phenylpyrazole-4-carbonitrile **65** with formic acid (Scheme 78) [116].

**Scheme 79:**
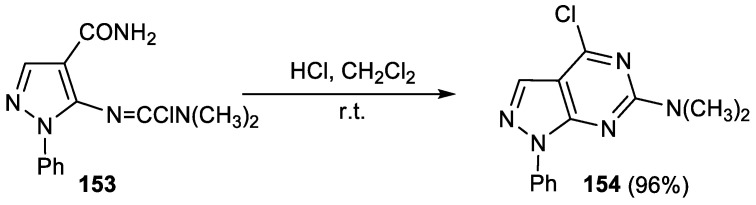
Cyclization of chloroamidine **154**.

The chloroamidine **153** was cyclized, in the presence of hydrochloric acid, to pyrazolo[3,4-*d*]pyrimidines **154** (Scheme 79) [117].

**Scheme 80:**
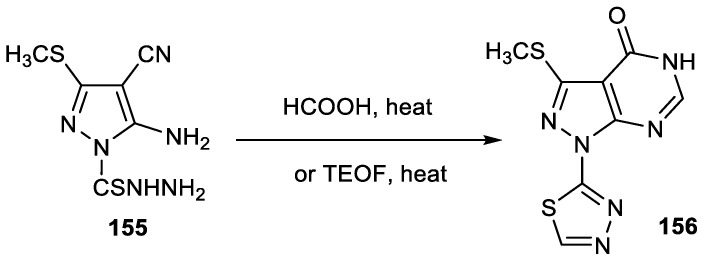
Synthesis of pyrazolo[3,4-*d*]pyrimidin-4(5*H*)-one **156**.

When pyrazolylcarbothiohydrazide **155** was treated with formic acid or triethyl orthoformate, it gave 3-methylsulfanyl-1-(1,3,4-thiadiazolyl-2-yl)pyrazolo[3,4-*d*]pyrimidin-4(5*H*)-one **156** (Scheme 80) [118].

**Scheme 81:**
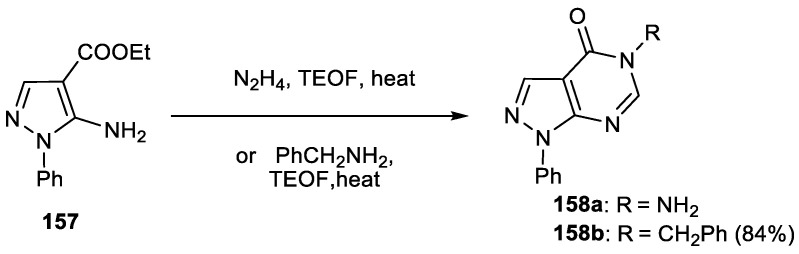
Synthesis of 5-substituted pyrazolo[3,4-*d*]pyrimidin-4(5*H*)-ones **158a**,**b**.

Ghorab et al. [114] reacted ethyl 5-amino-1-phenyl-1*H*-pyrazole-4-carboxylate (**157**) with hydrazine hydrate or benzyl amine in the presence of triethyl orthoformate (TEOF) to obtain the 5-substituted derivatives **158a**,**b** (Scheme 81) [114].

**Scheme 82:**
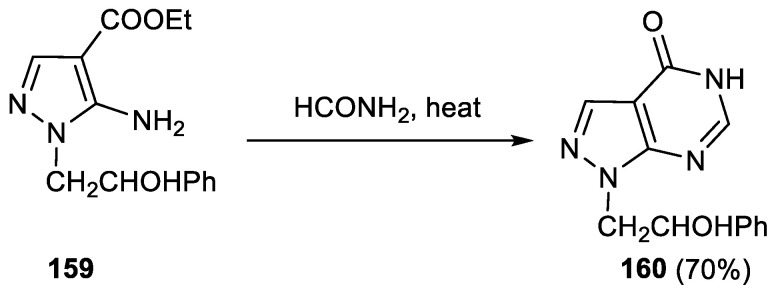
Synthesis of pyrazolo[3,4-*d*]pyrimidin-4-one **160**.

Fusion of **159** with formamide at 200 °C for 8 h afforded the corresponding pyrazolo[3,4-*d*]pyrimidin-4-one **160** (Scheme 82) [119].

**Scheme 83:**
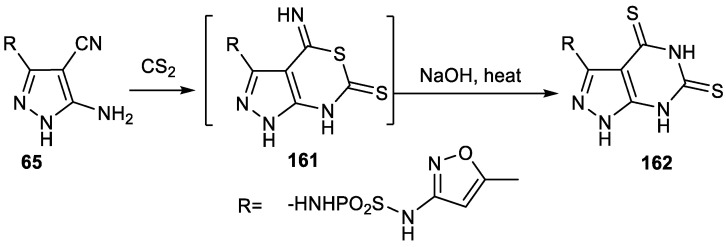
Synthesis of 4,6-dithioxopyrazolo[3,4-*d*]pyrimidine derivatives **162**.

5-Amino-1*H*-pyrazol-4-carbonitrile derivative **65** afforded with carbon disulfide in pyridine 4,6-dithioxopyrazolo[3,4-*d*]pyrimidine derivative **162** upon gentle refluxing. The reaction underwent a rearrangement process of the thiazine intermediate **161** during treatment with NaOH (Scheme 83) [120].

#### 3.1.3. Pyrazolopyridines

**Figure 8:**
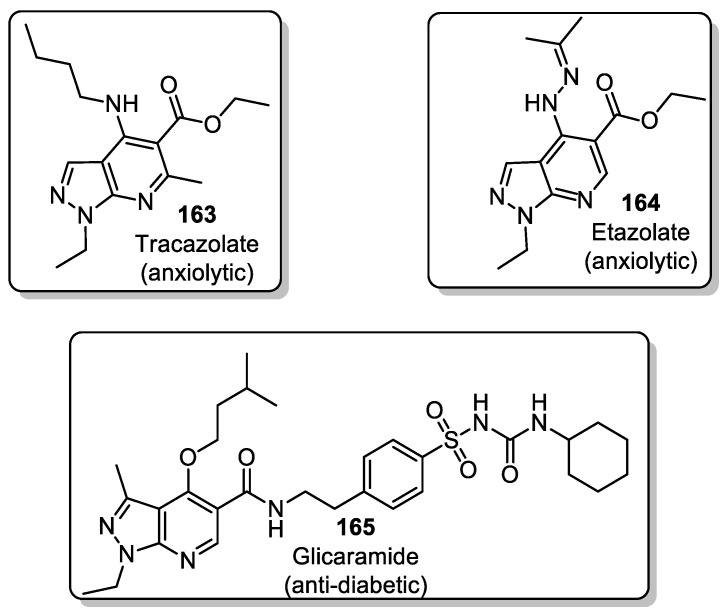
Drugs containing pyrazolo[3,4-*b*]pyridine scaffold **163**–**165**.

The pyrazolo[3,4-*b*]pyridine moiety is known as a privileged structural motif of drug-like molecules. Tracazolate **163**, etazolate **164**, and glicaramide **165** are considered as drug-analogous containing pyrazolo[3,4-*b*]pyridine (Figure 8) [121,122,123].

**Scheme 84:**
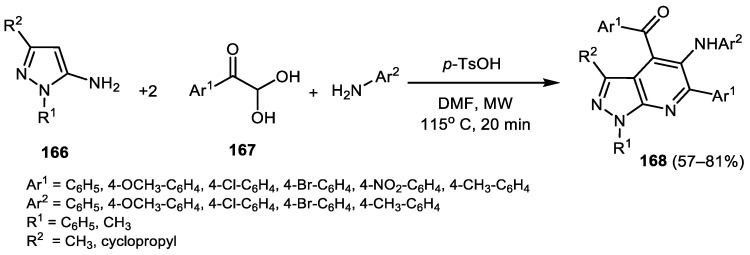
Synthesis of pyrazolo[3,4-*b*]pyridines **168**.

Jiang and co-workers [124] reported on microwave irradiation of a multicomponent reaction of 5-amino-pyrazoles **166** with arylglyoxal monohydrates **167** and aromatic amines in the presence of *p*-TsOH/DMF produced substituted acyl pyrazolo[3,4-*b*]pyridines **168** in good yields (Scheme 84) [124].

**Scheme 85:**
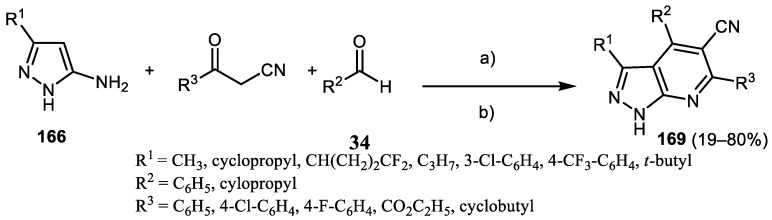
Synthesis of substituted pyrazolo[3,4-*b*]pyridine derivatives 169. Reagents and conditions; (a) Et_3_N (2 eq), DMF, 90 °C; (b) NaNO_2_ (3 eq) AcOH, rt.

One-pot synthesis of fully substituted 1*H*-pyrazolo[3,4-*b*]pyridines **169** was established based on a three-component approach between 5-aminopyrazoles **166**, *β*-ketonitriles, and aromatic/aliphatic aldehydes **34** in the presence of triethylamine (Scheme 85) [125].

**Scheme 86:**
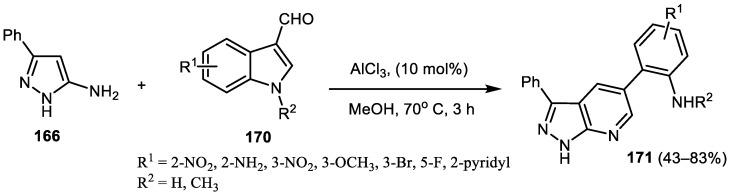
Synthesis of aryl pyrazolo[3,4-*b*]pyridines **171**.

Lee and Park [126] reported on the synthesis of aryl pyrazolo[3,4-*b*]pyridines **171** from 5-aminopyrazoles **166** and indole-3-carboxaldehydes **170** catalyzed by AlCl_3_ by the indole ring-opening without using catalysis with transition metals (Scheme 86) [126].

**Scheme 87:**
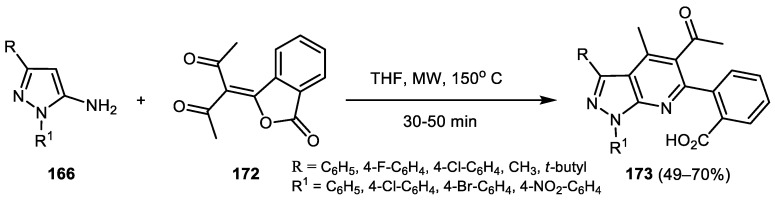
Synthesis of substituted pyrazolo[3,4-*b*]pyridines **173**.

In 2017, Portilla and co-workers [127] reported on the synthesis of substituted pyrazolo[3,4-*b*]pyridines **173** under microwave-assisted regioselective reaction to 5-aminopyrazoles **166** with 3-(3-oxo-2-benzofuran-1(3*H*)-ylidene)pentane-2,4-dione **172**. The reaction was based on a domino aza-Michael-cyclization-dehydration sequence (Scheme 87) [127].

**Scheme 88:**
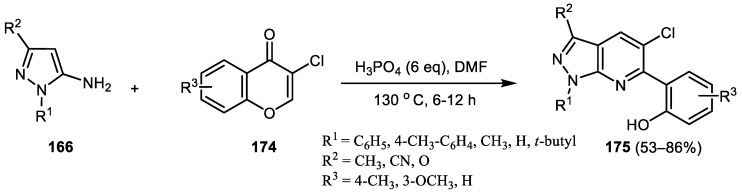
Synthesis of substituted pyrazolo[3,4-*b*]pyridines **175**.

An efficient facile synthesis of substituted pyrazolo[3,4-*b*]pyridines **175** in 53–86% yield was reported by Miliutina and co-workers [128]. The protocol was achieved by the reaction of 5-aminopyrazoles **166** with 3-chlorochromones **174** in the presence of phosphoric acid (Scheme 88) [128].

### 3.2. Heteroannulation with Five-Membered Heterocycles

#### 3.2.1. Imidazo-Pyrazole

**Scheme 89:**
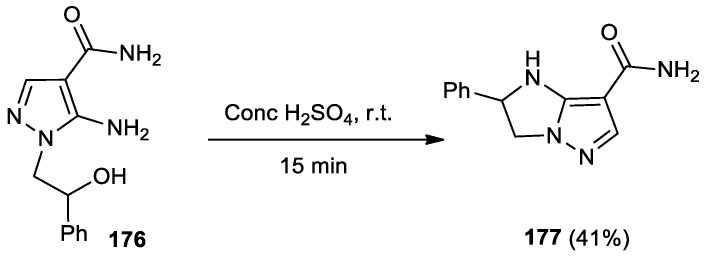
Synthesis of imidazo[1,2-*b*]pyrazole-7-carboxamide **177**.

2-Phenyl-2,3-dihydro-1*H*-imidazo[1,2-*b*]pyrazole-7-carboxamide **177** was prepared by cyclization of 5-amino-1-(2-hydroxy-2-phenylethyl)-1*H*-pyrazole-4-carboxamide **176** in the presence of concentrated sulfuric acid (Scheme 89) [129].

**Scheme 90:**
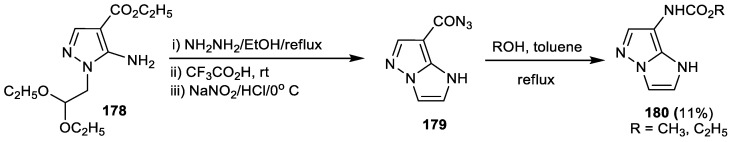
Synthesis of 7-substituted 1*H*-imidazo[1,2-*b*]pyrazoles **180**.

Ethyl 5-amino-1-(2,2-diethoxyethyl)-1*H*-pyrazole-4-carboxylate **178** was reacted with hydrazine followed by a reaction with nitrous acid to afford 1*H*-imidazo[1,2-*b*]pyrazole-7-carbonyl azide **179** rearranged to produce carbamates **180** (Scheme 90) [130].

**Scheme 91:**
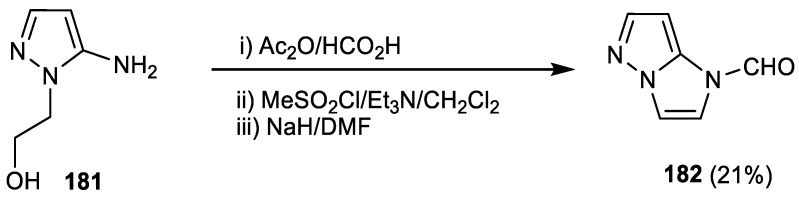
Synthesis of 1-formyl-imidazo[1,2-*b*]pyrazole **182**.

Amino-l-(2-hydroxyethyl)pyrazole **181** was formylated, treated with methanesulfonyl chloride and triethylamine, followed by cyclization with sodium hydride, to give 1-formyl-2,3-dihydro-1*H*-imidazo[1,2-*b*]pyrazole **182** (Scheme 91) [131].

**Scheme 92:**
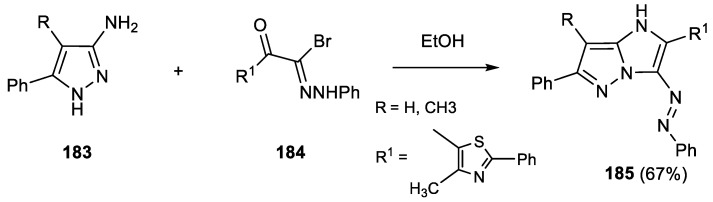
Synthesis of imidazo[1,2-*b*]pyrazoles **185**.

The 3-amino-5-phenylpyrazoles **183** reacted with 2-(4-methyl-2-phenyl-1,3-thiazol-5-yl)-2-oxo-*N*-phenylethanehydrazonoyl bromide **184** in boiling ethanol to give 3-phenylazo-2-(4-methyl-2-phenyl-thiazol-5-yl)-6-phenyl-5*H*-imidazo[1,2-*b*]pyrazoles **185** (Scheme 92) [132].

**Scheme 93:**
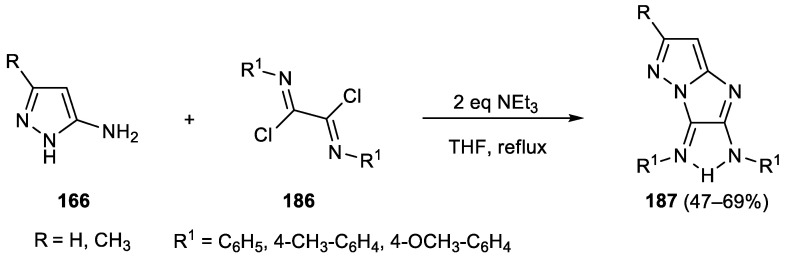
Synthesis of 3*H*-imidazo[1,2-*b*]pyrazoles **187**.

Regioselective cyclization reaction between compound **166** and oxaldiimidoyl dichlorides **186** in THF in the presence of triethylamine afforded 3*H*-imidazo[1,2-*b*]pyrazoles **187** in good yields (Scheme 93) [133].

**Scheme 94:**
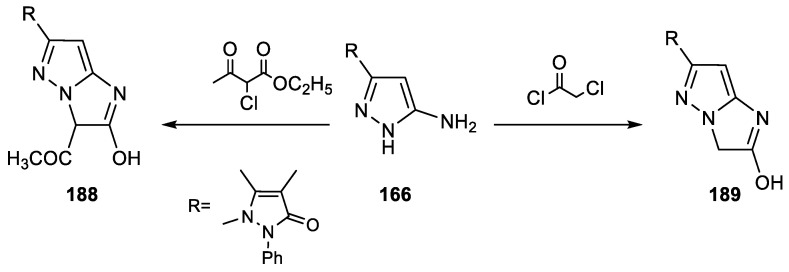
Synthesis of imidazo[1,2-*b*]pyrazole derivatives **188** and **189**.

5-Aminopyrazole **166** was reacted with either ethyl α-chloroacetoacetate or chloroacetyl chloride to yielded 1-(2-hydroxy-3*H*-imidazo[1,2-*b*]pyrazole-3-yl)ethanone **188** and 3*H*-imidazo[1,2-*b*]pyrazole-2-ol **189**, respectively (Scheme 94) [134].

**Scheme 95:**
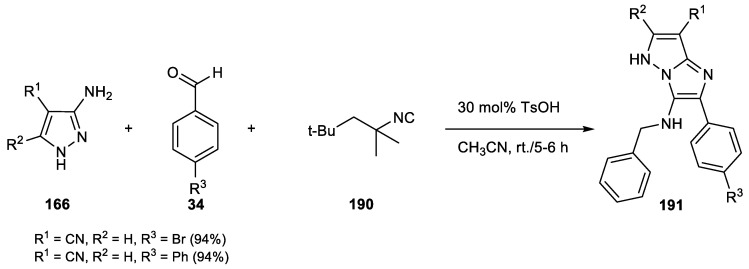
Synthesis of *N*-alkyl-2-aryl-5*H*-imidazo[1,2-*b*]pyrazole-3-amines **191**.

A series of *N*-alkyl-2-aryl-5*H*-imidazo[1,2-*b*]pyrazole-3-amines **191** in good to high yields were synthesized by the three-component condensation of aromatic aldehydes **34**, amino-pyrazole **166**, and isocyanide **190** in acetonitrile in the presence of 4-toluenesulfonic acid as a catalyst at room temperature (Scheme 95) [135].

#### 3.2.2. Thienopyrazoles

**Figure 9:**
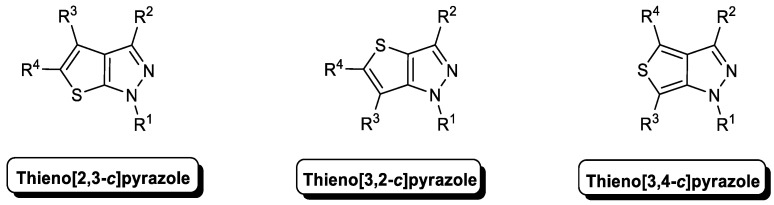
Regioisomers of thienopyrazoles.

There are three different regioisomers of thienopyrazoles, as shown in Figure 9.

**Scheme 96:**
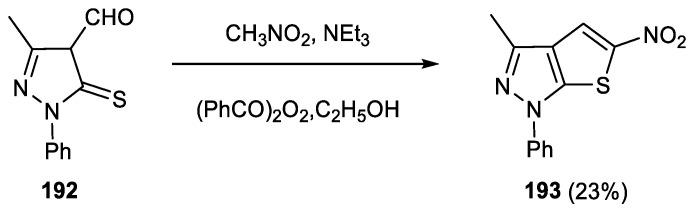
Synthesis of 5-nitro-3-methyl-1-phenylthieno[2,3-*c*]pyrazole **193**.

4,5-Dihydro-3-methyl-1-phenyl-5-thioxo-1*H*-pyrazole-4-carboxaldehyde **192** reacted with nitromethane in the presence of dibenzoyl peroxide, Et_3_N in ethanol to produce 5-nitro-3-methyl-1-phenylthieno[2,3-*c*]pyrazole **193** (Scheme 96) [136].

**Scheme 97:**
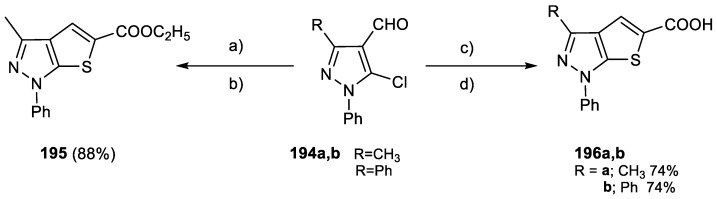
Synthesis of thieno[2,3-*c*]pyrazoles **195** and **196a**,**b**. Reagents and conditions; (a) HS-CH2-COOEt; (b) NaOEt/EtOH; (c) HS-CH2-COOMe; (d) 1-Me-ONa, MeOH, 60 °C, 2-NaOH, MeOH, reflux.

5-Chloro-3-methyl (or phenyl)-1-phenyl-1*H*-pyrazole-4-carbaldehyde **194a**,**b** reacted with ethyl thioglycolate in ethanol and presence of sodium ethoxide to give ethyl thienopyrazole carboxylate **195**. In a similar procedure, compounds **196a**,**b** were prepared after saponification with methanolic sodium hydroxide (Scheme 97) [137].

**Scheme 98:**
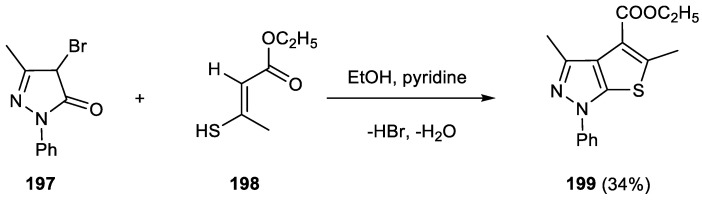
Synthesis of thieno[2,3-*b*]pyrazole **199**.

4-Bromo-3-methyl-1-phenyl-2-pyrazolin-5-one (**197**) reacted with ethyl 3-mercaptocrotonate (**198**) in an equimolar ratio in ethanol to afford thieno[2,3-*b*]pyrazole **199** (Scheme 98) [138].

**Scheme 99:**
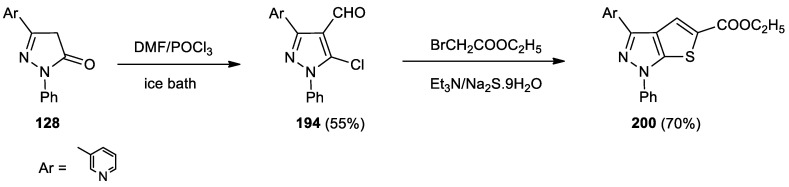
Synthesis of thieno[2,3-*c*]pyrazole-5-carboxylic acid ethyl ester **200**.

1-Phenyl-3-(pyridin-3-yl)-1*H*-thieno[2,3-*c*]pyrazole-5-carboxylic acid ethyl ester (**200**) was synthesized by the reaction of 5-chloro-1-phenyl-3-(pyridin-3-yl)-1*H*- pyrazole-4-carbaldehyde **194** with ethyl bromoacetate and sodium sulfide. First, reaction of 2-phenyl-5-pyridin-3-yl-2,4-dihydro-pyrazol-3-one (**128**) with Vilsmeier–Haack reagent gave **194** in 55% yield (Scheme 99) [139].

**Scheme 100:**
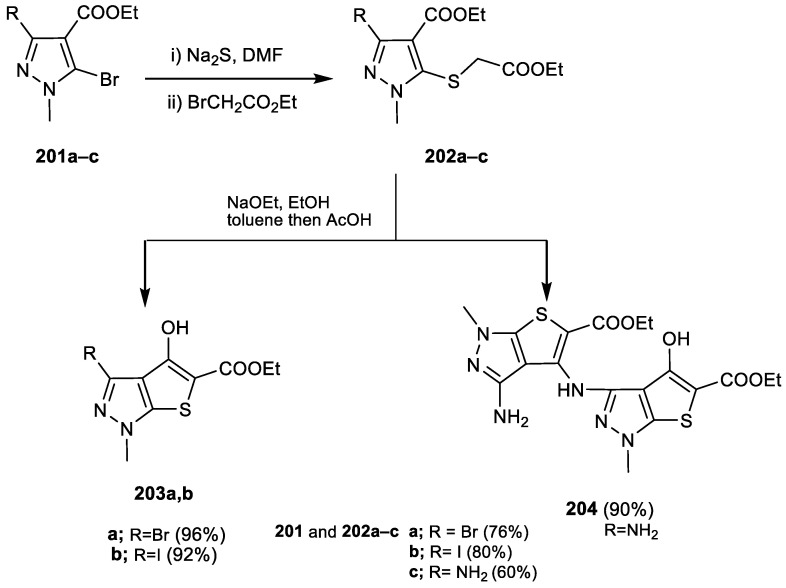
Synthesis of thieno[2,3-*c*]pyrazoles **203a**,**b** and **204**.

A practical and straightforward synthesis of 1-methyl-1*H*-thieno[2,3-*c*]pyrazoles from 3-amino-1*H*-pyrazole-4-carboxylic acid ethyl ester were reported by Toto et al. The 3-substituted ethyl pyrazole-5-sulfonylacetate derivatives **202a**–**c** were synthesized by the reaction of 3-substituted ethyl 5-bromo-*N*-methyl-pyrazole-4-carboxylates **201a**–**c** with ethyl bromoacetate and sodium sulfide in DMF. Base-catalyzed cyclization of the *S*-alkylated pyrazoles **202a**,**b** was accomplished using sodium ethoxide in toluene to afford the expected ethyl 4-hydroxythieno[2,3-*c*]pyrazole-5-carboxylate derivatives **203a**,**b**. Moreover, cyclization of the amine analog **202c** under the same conditions yielded the imine derivative **204**, which probably came from the self-condensation of the expected amino-thieno fused compound **202c** (Scheme 100) [140].

**Scheme 101:**
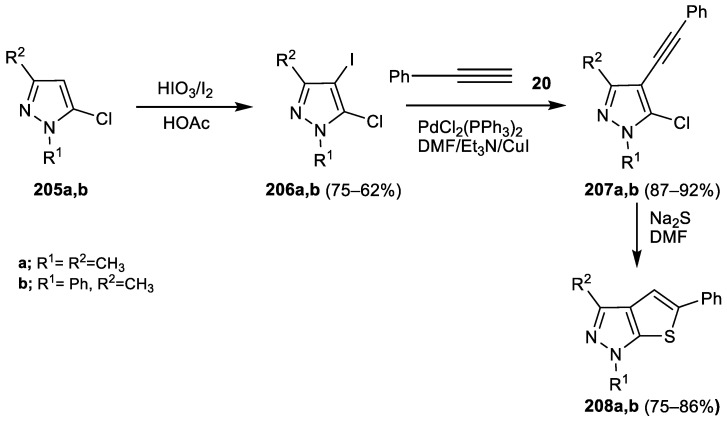
Sonogashira coupling method to synthesize thieno[2,3-*c*]pyrazoles **208a**,**b**.

Using the Sonogashira coupling method and starting with pyrazole derivatives to synthesize thieno[2,3-*c*]pyrazole was reported by Eller et al. [141]. The strategy depends upon the treatment of the available 1,3-disubstituted-5-chloro-1*H*-pyrazoles **205a**,**b** with I_2_–HIO_3_ to obtain the corresponding 5-chloro-4-iodopyrazoles **206a**,**b**. The latter compounds were selectively connected to phenylacetylene (**20**) by a Sonogashira cross-coupling reaction, yielding only the 4-(phenylethynyl)pyrazoles **207a**,**b** in good yields (87–92%). The final reaction step, compounds **207a**,**b,** was then subjected to sodium sulfide in DMF to produce compounds **208a**,**b** (Scheme 101) [141].

**Scheme 102:**
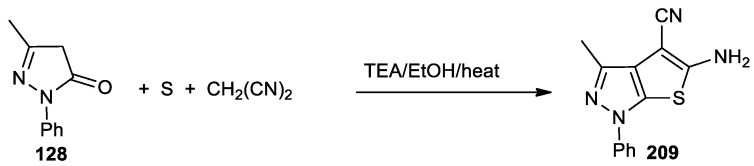
Synthesis of amino cyano thieno[2,3-*c*]pyrazole **209**.

Sabaa et al. and Rabie et al. [142,143] have synthesized thieno[2,3-*c*]pyrazole **209** using the Gewald reaction. The *N*-phenyl pyrazolone **128** underwent the Gewald reaction and reacted with sulfur and malononitrile in equimolar ratios under reflux for 3 h in the presence of triethyl amine (TEA) and absolute ethanol as a solvent to give the amino cyano derivative of thienopyrazole **209** (Scheme 102) [142,143].

**Scheme 103:**
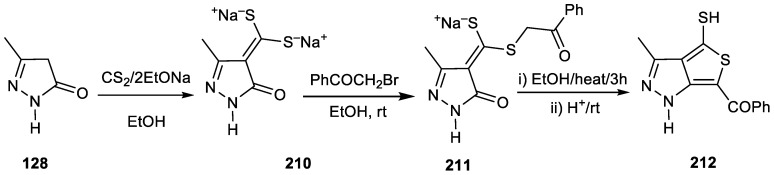
Synthesis of thienopyrazole-4-thiol **212**.

Elgemeie et al. [144] reported the synthesis of thieno[3,4-*c*]pyrazole ring system **212** (Scheme 103). Preparation of **212** started through the reaction of pyrazolin-5-one **128** reacted carbon disulfide in the presence of sodium ethoxide to afford the sodium dithiolate **210**. Then, one equivalent of phenacyl bromide was added to **210** to give the corresponding sodium salt of monoalkylated product **211**. Finally, compound **211** was cyclized to afford the thienopyrazole-4-thiol **212** upon refluxing with sodium ethoxide, followed by acidification (Scheme 103) [144].

**Scheme 104:**
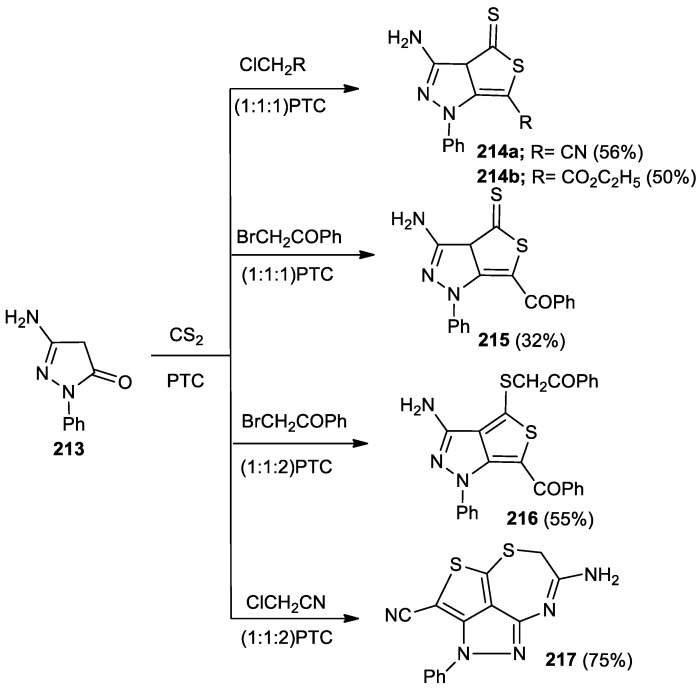
Synthesis of thieno[3,4-*c*]pyrazoles **214**–**217**.

El-Saraf et al. [145] prepared a series of thieno[3,4-*c*]pyrazoles via reaction of the 3-aminopyrazolin-5-one **213** with CS_2_ and different molar ratios of various halo compounds having active methylene under phase transfer condition (PTC), which afforded compounds **214**–**217** (Scheme 104) [145].

#### 3.2.3. Furopyrazole

**Scheme 105:**
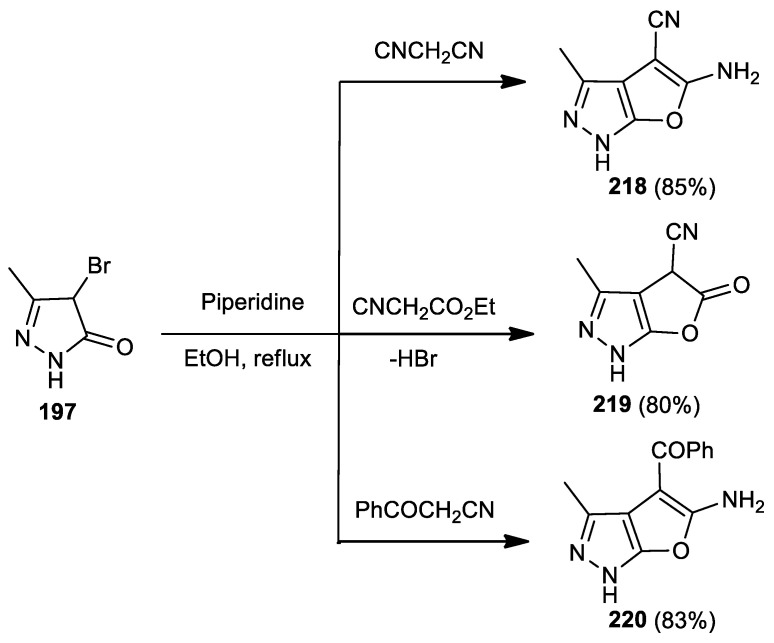
Synthesis of furo[2,3-*c*]pyrazoles **218**–**220**.

Furopyrazoles are known to have antitumor, antiproliferative, and antimicrobial activities. Aziz et al. observed that equimolecular amounts of 3-methyl-4-bromo-2-pyrazolin-5-one (**197**) and malononitrile reacted in absolute ethanol in the presence of piperidine under reflux for 3 h to give furo[2,3-*c*]pyrazole **218** in 85% yield (Scheme 105) [146]. Then compound **197** reacted with ethyl cyanoacetate to give furo[2,3-*c*]pyrazole **219** in 80% yield. Whereas benzoylacetonitrile reacted with compound **197** to afford furo[2,3-c]pyrazole **220** in 83% yield (Scheme 105) [147].

**Scheme 106:**
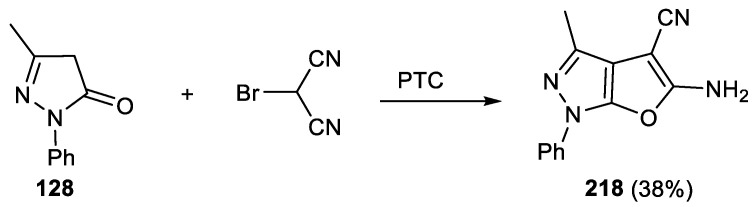
Synthesis of *N*-phenyl furo[2,3-*c*]pyrazole **218**.

Reaction of 3-methyl-1-phenyl-pyrazol-5-one (**128**) with bromomalononitrile under PTC conditions [K_2_CO_3_/benzene/tetrabutyl ammonium bromide (TBAB) catalyst] afforded 5-amino-4-cyano-3-methyl-*N*-phenyl-furo[2,3-*c*]pyrazole **218** in 38% yield (Scheme 106) [148]. The formation of compound **218** was assumed to involve HBr elimination followed by a nucleophilic attack of the OH group to electrophilic carbonitrile, followed by cyclization and aromatization (Scheme 106) [148].

**Scheme 107:**
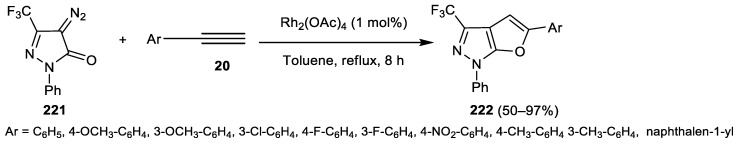
Synthesis of CF_3_-substituted ring-fused furo[2,3-*c*]pyrazoles **222**.

Rh_2_(OAc)_4_ was used as a catalyst of [3 + 2]cycloaddition reaction between 4-diazo-1-phenyl-3-(trifluoromethyl)-1*H*-pyrazol-5(4*H*)-one **221** and aromatic alkynes **20** (Scheme 107) [149].

**Scheme 108:**
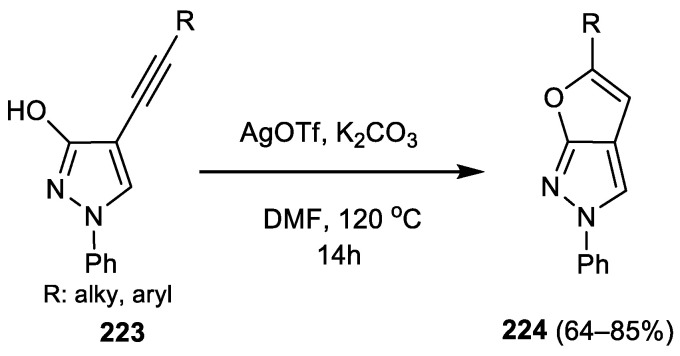
Synthesis of 2*H*-furo[2,3-*c*]pyrazoles **224**.

In 2019, Milišiūnaitė et al. [150] reported that the synthesis of 2*H*-furo[2,3-*c*]pyrazoles **224** was achieved 5-endo-dig cyclization to afford 4-alkynyl-3-hydroxy-1-phenyl-1*H*-pyrazoles **223** as a key step and catalyzed by AgOTf/K_2_CO_3_. The reactions were complete in DMF at 120 °C after 14 h, and the products **224** were obtained in 64–85% yields (Scheme 108) [150].

**Scheme 109:**
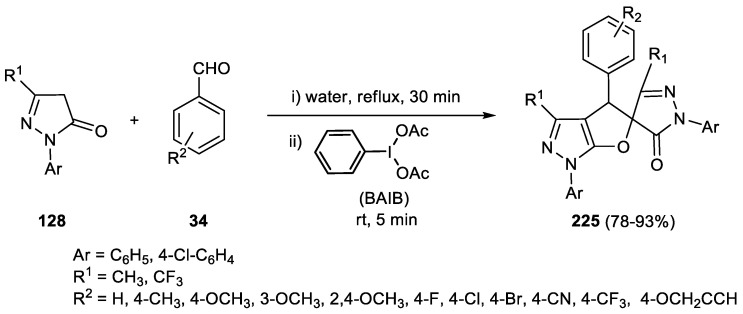
Synthesis of dihydrospirofuro[2,3-*c*]pyrazoles **225**.

Synthesis of dihydrospirofuro[2,3-*c*]pyrazoles **225** was reported by Kale et al. [151] from the reaction of pyrazolones **128** with aldehydes (**34**) in boiling water for 30 min followed by addition of bis(acetoxy)-iodobenzene at room temperature for 5 min (Scheme 109) [151].

**Scheme 110:**
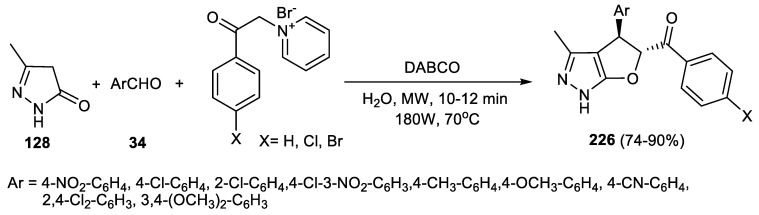
Synthesis of furo[2,3-*c*]pyrazole derivatives **226**.

4,5-Dihydro-1*H*-furo[2,3-*c*]pyrazole derivatives **226** were synthesized by a one-pot domino reaction involving pyrazolone **128**, aromatic aldehydes **34**, and a pyridinium salt catalyzed by DABCO with high diastereoselectivity in H_2_O under microwave irradiation (Scheme 110) [152].

**Scheme 111:**
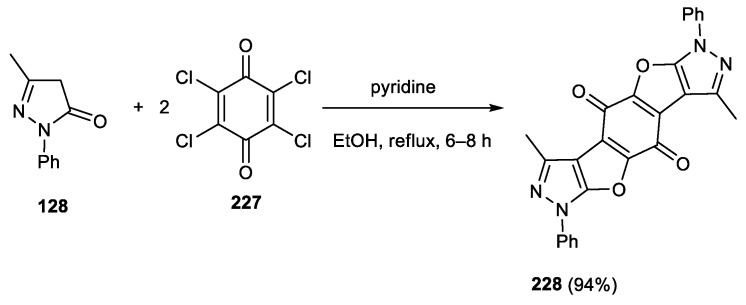
Synthesis of benzo[2,3-*b*;2′,3′-*b*]bis-furo[3,2-*d*]pyrazole-5,10-dione **228**.

Reaction of **128** with *p*-chloranil (**227**) in the presence of pyridine in EtOH at reflux for 6–8 h afforded 4,9-dimethyl-2,7-diphenyl-benzo[2,3-b;2′,3′-b]bisfuro[3,2-*d*]pyrazole-5,10-dione (**228**) in 94% yield (Scheme 111) [153].

## 4. Biological Activities

### 4.1. Anticancer Activity

**Figure 10:**
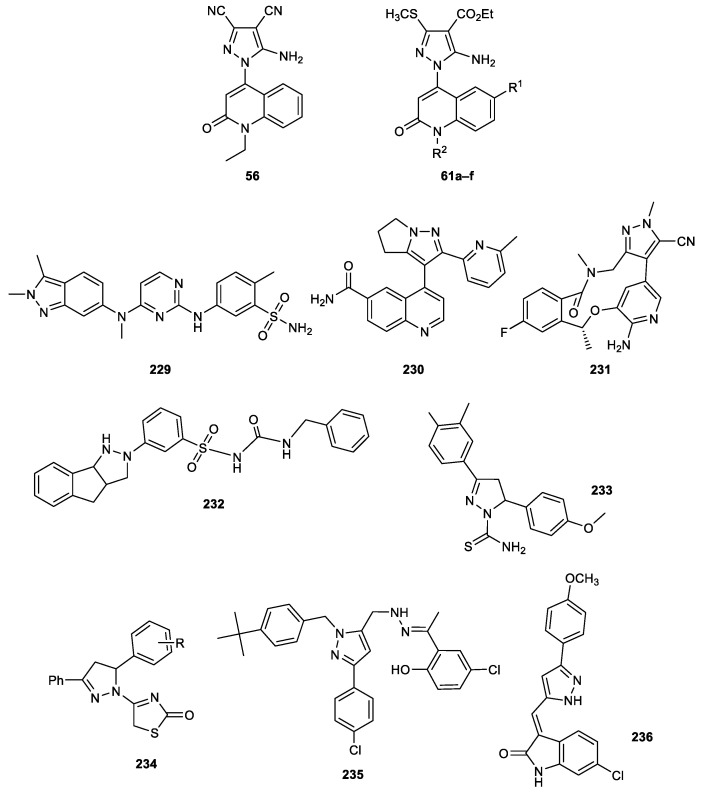
Structures of some anticancer active pyrazoles **56**, **61a**–**f**, and **229**–**236**.

It was previously mentioned by Aly et al. (see Scheme 23) [60] that 5-amino-1-(1-ethyl-2-oxo-1,2-dihydroquinolin-4-yl)-1*H*-pyrazole-3,4-dicarbonitrile **56** showed a good antiproliferative EGFR-TK inhibition activity against many tumor cell lines. Moreover, a series of pyrazole/quinolones **61a**–**f** (Figure 10) showed remarkable anticancer activities [61]. Compounds **61a**, **61c**, and **61f** showed a significant decrease in inflammatory mediators TNFα and CRB greater than NAC when compared to model group exhibited a significant decrease in comparison to NAC, especially compound **61c** whose found CRB conc 1.90 (mg/dL) in comparison to NAC of conc 2.13 mg/dL.

In 2016, Wu, P.; reported that 5-((4-((2,3-dimethyl-2*H*-indazol-6- yl)(methyl)amino)pyrimidin-2-yl)amino)-2-methylbenzene-sulfonamide (Figure 10, **229**) as molecule kinase inhibitor [154].

Galunisertib (Figure 10) is known as 6-quinoline carboxamide of pyrazole derivative **230** [155], and it is an oral drug that is described as an available, small molecule antagonist of the tyrosine kinase transforming growth factor-beta (TGF-β) receptor type 1 (TGFBR1), with potential antineoplastic activity.

Another pyrazolo-anticancer drug known as Lorlatinib **231** (Figure 10) [156] is an orally available drug known as ATP-competitive inhibitor of the receptor tyrosine kinases, anaplastic lymphoma kinase (ALK), and C-ros oncogene 1 (Ros1), with potential antineoplastic activity. Lorlatinib binds to and inhibits both ALK and ROS1 kinases. The kinase inhibition leads to disruption of ALK- and ROS1-mediated signaling and eventually inhibits tumor cell growth in ALK- and ROS1-overexpressing tumor cells.

Al-Saadi et al. [157] synthesized a series of pyrazole and pyrazoline **232** fused ring systems substituted with anticancer biologically active chemical species. Lv et al. [158] synthesized a series of pyrazole-1-carbothioamide derivatives that showed high antiproliferative activity against MCF-7 with IC_50_ 0.08 μM. Among them, compound 3-(3,4-dimethylphenyl)-5-(4-methoxyphenyl)-4,5-dihydro-1*H*-pyrazole-1-carbothioamide **233** is most potent with IC_50_ of 0.07 μM, as compared to positive control erlotinib (IC_50_ of 0.03 μM) [158].

The anticancer activity of several thiazolone-based compounds containing the 5-aryl-3-phenyl-4,5-dihydro-1*H*-pyrazol-1-yl **234** was examined by Havrylyuk et al. (Figure 10) [159]. Whereas Zheng et al. synthesized a series of 3-aryl-1-(4-*tert*-butylbenzyl)-1*H*-pyrazole-5-carbohydrazidehydrazone derivatives and investigated their effects on A549 cell growth, the compound (*E*)-2-(1-(2-((1-(4-(*tert*-butyl)benzyl)-3-(4-chlorophenyl)-1*H*-pyrazol-5-yl)methyl)hydrazono)ethyl)-4-chlorophenol **235** (Figure 10) showed high growth inhibitory effect and induced apoptosis of A549 lung cancer cells [160]. On the other hand, Kamal et al. reported the synthesis of oxindole–pyrazole derivatives as potent microtubules binders/anticancer agents. Among all, compound **236** (Figure 10) showed anti-proliferative agents with average IC_50_ = 3 μM against HeLa, A549, MCF7, and DU145 cancer cell lines compared to the reference drug nocodazole with average IC_50_ = 1.72 μM [161].

**Scheme 112:**
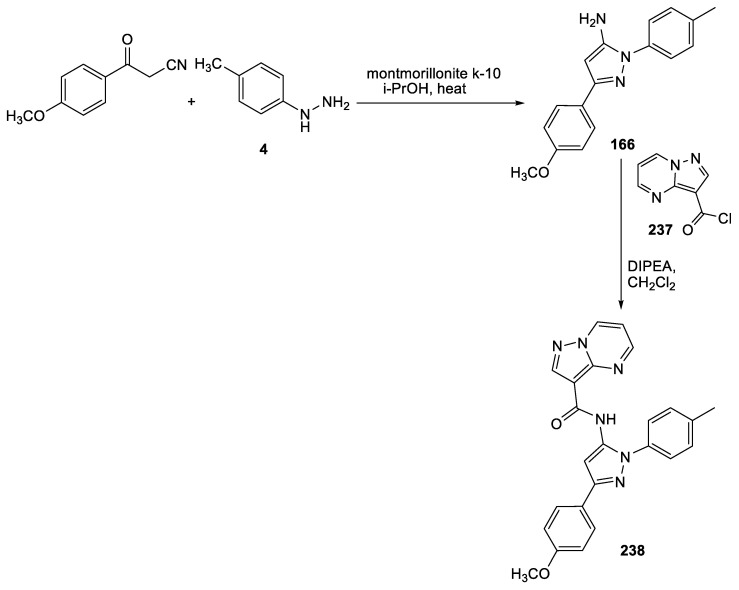
Synthesis of pyrazole IRAK4 inhibitor **238**.

Inhibitor **238** was synthesized by McElroy et al. [162]. The reaction of the pyrazole **166** with pyrazolo[1,5-*a*]pyrimidine-3-carbonyl chloride (**237**) in the presence of *N*, *N*-diisopropylethylamine, or Hünig’s base (DIPEA), produced a series of potent, selective, and orally pyrazole interleukin receptor-associated kinase4 (IRAK4), as shown in Scheme 112 [162].

Lim et al. reported on synthesizing a series of 5-amino-*N*-(1*H*-pyrazol-4-yl)-pyrazolo-[1,5-*a*]pyrimidine-3-carboxamides **239** and **240** as IRAK4 inhibitors.

**Figure 11:**
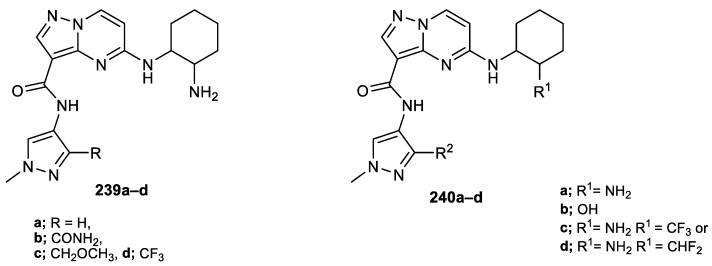
Structures of some IRAK4 inhibitors pyrazoles **239** and **240**.

Different substituents of **239** and **240** led to identifying IRAK4 inhibitors with excellent potency, kinase selectivity, and pharmacokinetic properties suitable for oral dosing (Figure 11) [163].

### 4.2. Monoamine Oxidase Inhibitors

**Figure 12:**
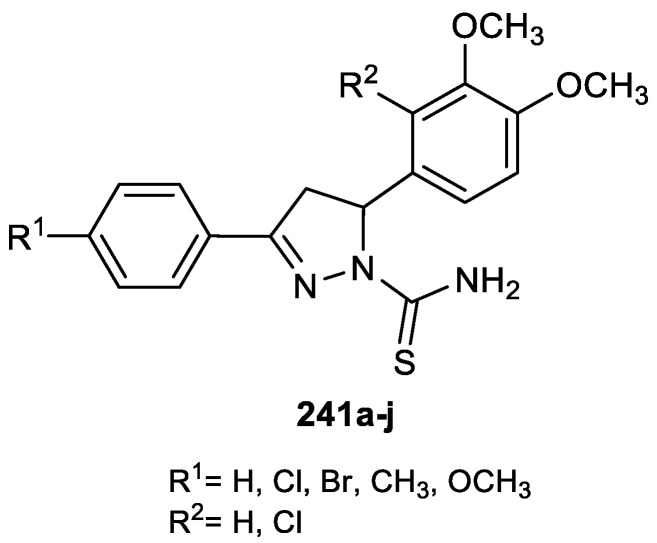
Structures of some IRAK4 inhibitors pyrazoles **241a**–**j**.

Palaska et al. reported on synthesizing several *N*^1^-thiocarbamoyl-3,5-diaryl- 4,5-dihydro-(1*H*)-pyrazoles **241a**–**j**. The obtained compounds were screened as monoamine oxidase (MAO) inhibitors against monoamine oxidases isolated and purified from the mitochondrial extracts of rat liver homogenates and human platelets (Figure 12) [164].

### 4.3. Antimicrobial and Antifungal Activity

**Figure 13:**
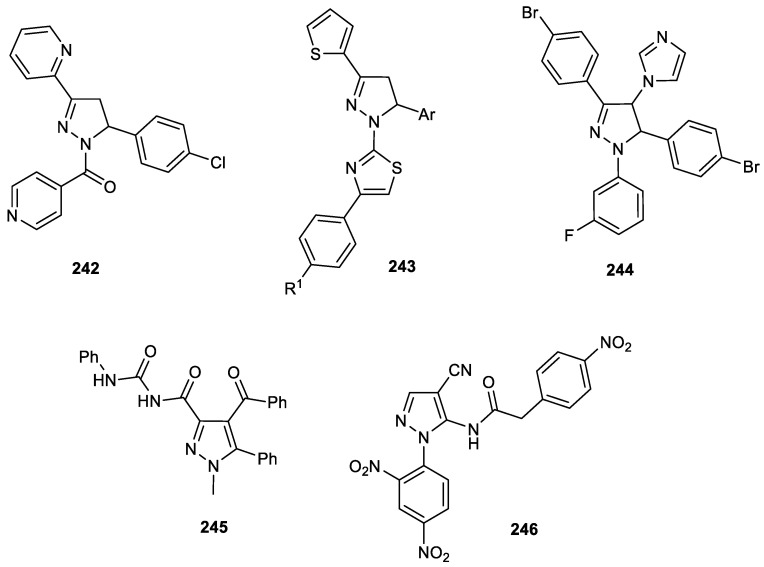
Structures of some antibacterial active pyrazoles **242**–**246**.

5–Aryl-isonicotionyl-3-(pyridine-2-yl)-4,5–dihydro-1*H*-pyrazoles **242** (Figure 13) were synthesized and showed significant antimycobacterial activity [165].

Özdemir et al. prepared several series of 1-(4-aryl-2-thiazolyl)-3- (2-thienyl)-5-aryl-2-pyrazoline derivatives **243** (Figure 13) and screened them for antimicrobial activities against, e.g., *Escherichia coli*, *Staphylococcus aureus*, *Salmonella typhimurium*, *Bacillus cereus*, *Streptococcus faecalis*, *Aeromonas hydrophila*, *Candida albicans,* and *Candida glabrata* [166].

Zampieri et al. synthesized several 1-(3,5-diaryl-4,5-dihydropyrazol-4-yl)-1*H*–imidazole derivatives **244** (Figure 13) and tested for their in vitro antifungal and antimycobacterial activities. These imidazole derivatives showed excellent antifungal activity against the clinical strain of *C. albicans* [167].

Akbas et al. prepared 4-benzoyl-1-methyl-5-phenyl-*N*-(phenylcarbamoyl)-1*H*-pyrazole-3-carboxamide (**245**) activity against *Bacillus cereus*, *Staphylococcus aureus*, *Escherichia coli*, and *Pseudomonas putida*. The results showed that compound **245** (Figure 13) exhibits good antibacterial activity against Gram-positive and Gram-negative bacteria [168]. A series of 5-amido-1-(2,4-dinitrophenyl)-1*H*-pyrazole-4-carbonitriles was reported by Rahimizadeh et al., showing that compound **246** exhibited antimicrobial activities against *methicillin-*susceptible, *Staphylococcus* *aureus* (MSSA), and *methicillin-resistant Staphylococcus* *aureus* (MRSA), with MIC values of 25.1 µM [169].

**Figure 14:**
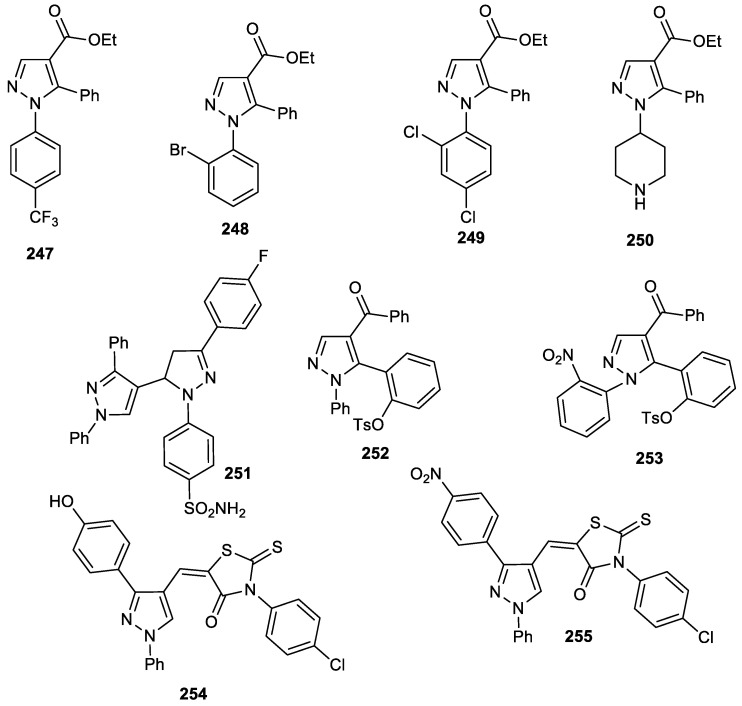
Structures of some antibacterial active pyrazoles **247**–**255**.

A series of pyrazole derivatives were synthesized and screened as antibacterial agents against *Staphylococcus aureus*, *Bacillus subtilis*, *Escherichia* *coli,* and *Pseudomonas aeruginosa*. Among the tested compounds, **247**–**250** (Figure 14) indicated excellent antibacterial activity against all the tested bacterial strains as compared with the standard drug ceftriaxone, which was active at 3.125, 1.6125, 1.6125, and 1.6125 µg/mL against *Staphylococcus aureus*, *Bacillus su*btilis, *Escherichia* *coli*, and *Pseudomonas aerug*inosa strains, respectively (Figure 14) [170].

Compound **251** inhibits activity against both Gram-positive and Gram-negative bacteria [171]. In addition, pyrazole derivatives **252**–**253 (**Figure 14) were prepared and screened for their antibacterial and antifungal activities using ampicillin and norcadine as standard drugs. All compounds were screened for their antimicrobial activities [172].

3-(4-Chlorophenyl)-5-((1-phenyl-3-aryl-1*H*-pyrazol-4-yl)methylene)-2-thioxothiazolidin-4-ones were prepared by B’Bhatt and Sharma. Compound **254** (Figure 14), as a derivative of the last series, was found to show potent activity against *Escherichia* *coli*, while compound **255** (Figure 14) was found to be potent against *S. a*ureus, *S. py*ogenes, and was found to have very good activity against *Candida albicans* [173].

In 2020, Alnufaie et al. reported on the synthesis of series of naphthyl-substituted pyrazole-derived hydrazones **260** [174].

**Scheme 113:**
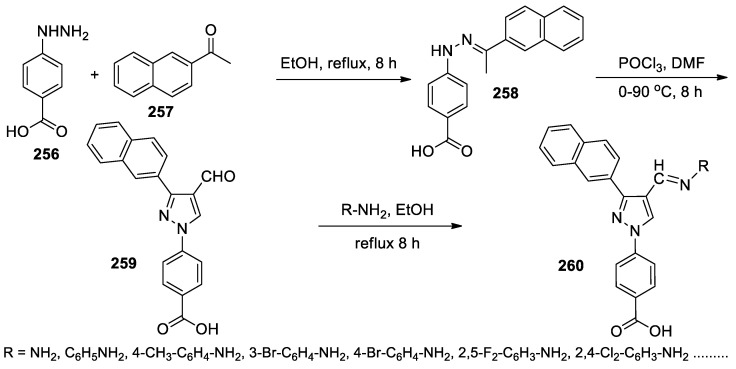
Synthesis of 4-[4-formyl-3-(2-naphthyl)pyrazol-1-yl] benzoic acid derivatives **260** as potent growth inhibitors of drug-resistant *Staphylococcus aureus* and *Acinetobacter baumannii*.

Reaction of 4-hydrazinobenzoic acid (**256**) with 2-acetylnaphthalene (**257**) afforded the corresponding condensed product **258**, which on Vilsmeier–Haack reagent gave compound **259**. Finally reaction of **259** with hydrazine derivatives produced the corresponding pyrazoles **260** (Scheme 113) [174]. Many of these pyrazoles showed potent growth inhibitory properties for planktonic *Staphylococcus aureus* and *Acinetobacter baumannii*, and its drug-resistant variants with MIC values as low as 0.78 and 1.56 μg/mL, respectively. These compounds also show potent activity against *Staphylococcus aureus* and *Acinetobacter baumannii* biofilm formation and eradication properties [174].

**Scheme 114:**
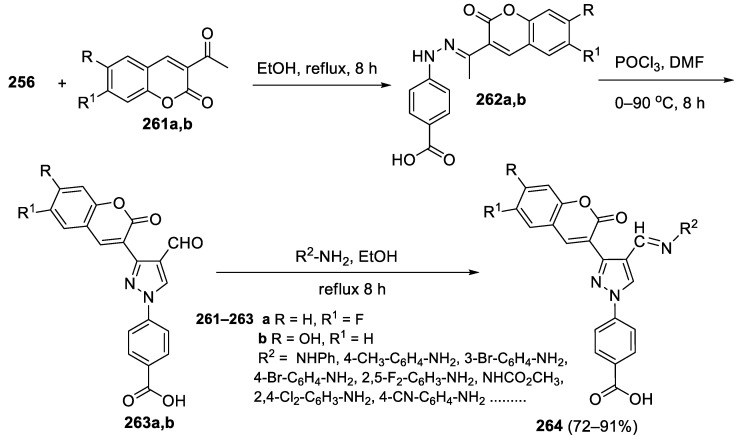
Synthesis of antibacterial pyrazole derivatives **264**.

Similarly, the same group published on the synthesis and antimicrobial studies of 31 coumarin-substituted pyrazole derivatives **264** [175]. The reaction of 4-hydrazinobenzoic acid **256** with fluoro **261a** and hydroxy **261b** substituted 3-acetylcoumarin formed the corresponding hydrazones **262a**,**b**, which were subjected to further reaction with POCl_3_/DMF to give the formyl-substituted pyrazole derivatives **363a**,**b** (Scheme 114). A series of hydrazone derivatives were then obtained via the reaction of **263a**,**b** with various hydrazine derivatives (Scheme 114) [175]. Some of these compounds have shown potent activity against methicillin-resistant *Staphylococcus aureus* (MRSA) with MIC as low as 3.125 μg/mL. These results are very significant, as MRSA strains have emerged as one of the most menacing pathogens of humans, and this bacterium is bypassing HIV (in terms of fatality rate). Some pyrazole derivatives inhibited the growth of cell lines with an IC_50_ around 15 μg/mL [175].

**Figure 15:**
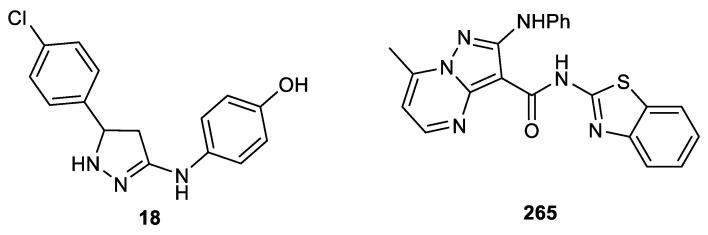
Structures of some inflammatory active pyrazoles **18** and **265**.

Sahu et al. also prepared 4-((5-(4-chlorophenyl)-4,5-dihydro-1*H*-pyrazol-3-yl)amino)phenol (**18**) (Figure 15), which showed antimicrobial activity and antibacterial activity. Antifungal activity was tested on Sabouraud Dextrose Agar plates by the cup–plate method against *Candida albicans* and *Aspergillus niger*. In both of these assays, ciprofloxacin and clotrimazole were used as standard drugs [46].

Bondock et al. reported on the synthesis of groups of pyrazole-pyrimidine derivatives. One of them, *N*-(benzo[*d*]thiazol-2-yl)-7-methyl-2-(phenylamino)pyrazolo-[1,5-*a*]pyrimidine-3-carboxamide **265**, was found to exhibit the most potent in vitro antifungal activity with MICs (6.25 μ/mL) against *A. fumigatus* and *F. Oxysporum,* comparable with cycloheximide (3.125 μ/mL) [176].

### 4.4. Anti-Inflammatory Activity

**Figure 16:**
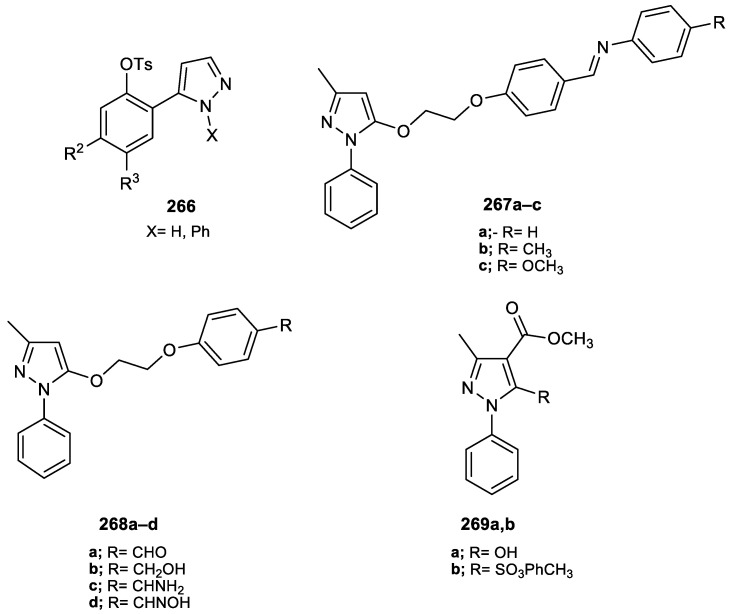
Structures of anti-inflammatory pyrazoles **266**–**269**.

Kendre et al. reported some 1*H*-pyrazole derivatives containing aryl sulfonate moieties **266** with anti-inflammatory effects (Figure 16) [177].

Tewari et al. prepared pyrazole derivatives **267**–**269**, and their anti-inflammatory activities were screened using carrageenan rat paw edema bioassay. Among the reported compounds, **268b** showed maximum COX-2 inhibitory potency (IC_50_ = 0.44) µM), while compounds **269a** and **269b** showed intermediate effects. (Figure 16) [178].

**Figure 17:**
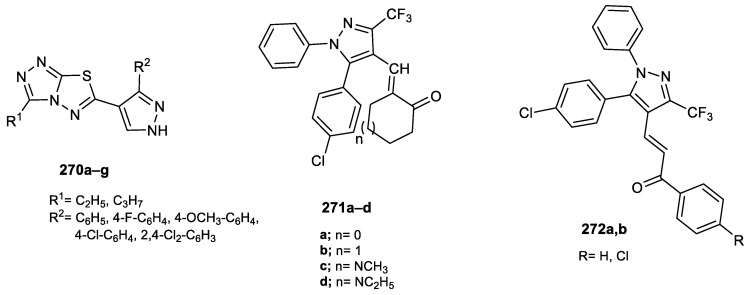
Structures of some inflammatory active pyrazoles **270**–**272**.

3,6-Disubstituted-1,2,4-triazolo[3,4-*b*]-1,3,4-thiadiazoles bearing pyrazole moieties **270a**–**g** (Figure 17) were screened as anti-inflammatory agents [179]. Among the reported compounds, compound **270g** showed the most significant anti-inflammatory activity (64.7% inhibition) compared to the standard drug diclofenac sodium (80.4% inhibition), whereas compounds **270d** and **270f** showed 56.9% inhibition. The propyl and *p*-chlorophenyl substituents of **270b** and **270f** showed significant activity. Whereas compounds have ethyl and *p*-chlorophenyl moieties, **270a** and **270c** accounted for moderate activities [179].

El-Sayed et al. also synthesized pyrazole derivatives **271** and **272** (Figure 17), and their anti-inflammatory activities were screened. Compounds **271a** and **271d** were found as the most selective among the tested compounds with good inhibitory profiles against COX-2 (Figure 17) [180].

### 4.5. Antiviral Activity

**Figure 18:**
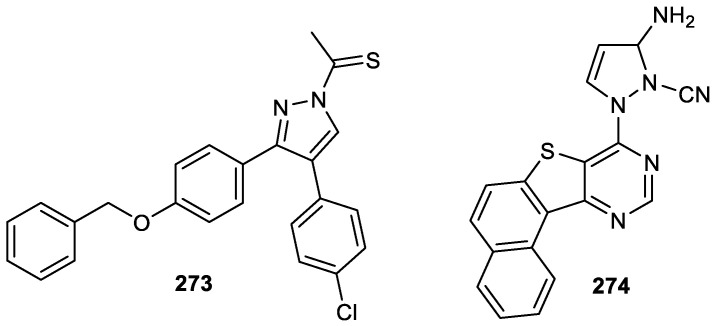
Structures of some antiviral active pyrazoles **273** and **274**.

It was reported that the derivative containing the R = Cl group of a series of 4,5-disubstituted pyrazole derivatives **273** (Figure 18) showed broad potent antiviral activity against a broad panel of viruses in different cells cultures (HEL Cell cultures) [181]. Moreover, substituted pyrazole derivatives **274** (Figure 18) showed good antiviral activity against hepatitis A [182].

### 4.6. Anti-Alzheimer’s Activity

**Figure 19:**
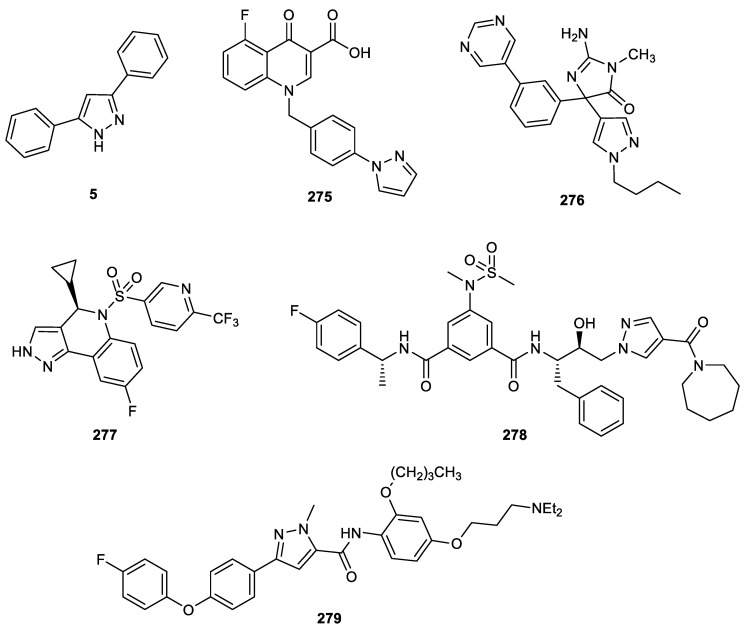
Structures of some pyrazoles **5** and **275**–**279** of anti-Alzheimer’s activity.

A series of 3,5-diaryl pyrazoles **5** (Figure 19) was assayed for their ability to inhibit monoamine oxidase-A (MAO-A) and monoamine oxidase B (MAO-B) reversibly. Several compounds show inhibitory activity with concentration values in the nanomolar range [183]. Kuduk et al. identified compound **275** (Figure 19) as a potent and selective full agonist of the M1 positive allosteric modulators [184]. In the same manner, compound **275** showed good inhibitory activity against MAO-A and MAO-B but low selectivity (IC_50_ MAO-A = 9.00 nM, IC_50_ MAO-B = 8.00 nM, and SI = 1.00).

A group of pyrazolyl and thienyl aminohydatoins was prepared by Malamas et al. and was tested as potent BACE1 inhibitors [185]. The *n*-butyl analog **276** was the most potent analog, with an IC_50_ value of 8 nM.

Zou et al. reported on the synthesis of a series of pyrazole-based compound **277** (Figure 19) and identified as *C*-terminus β-secretase 1 (BACE1) inhibitors [186]. Further, modification over the pyrazole scaffold leads to the identification of compound **278** as a potent inhibitor of BACE1 with an IC_50_ value of 0.025 µM.

Results reported by Han et al. indicated that the most active analogs **279** (Figure 19) exhibited higher inhibitory activities, with significant brain A *β*-lowering effects, as well as favorable aqueous solubility [187].

**Figure 20:**
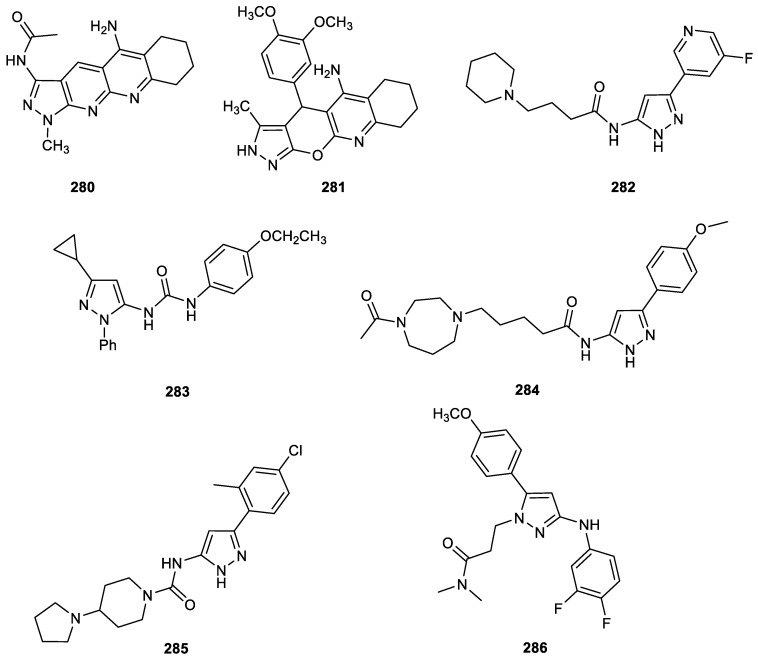
Structures of some pyrazoles **280**–**286** of anti-Alzheimer’s activity.

As acetylcholinesterase (AChE) inhibitors, pyrazolotacrines **280** (Figure 20) were reported by Silva et al. The results showed that compound **280** was the most potent inhibitor of AChE, which inhibited the enzyme above with an IC_50_ value of 0.069 µM [188]. Whereas Khoobi et al. synthesized compound **281** bearing 3,4-dimethoxyphenyl group was the most potent compound against acetylcholinesterase (AChE) [189], being more active than the reference drug tacrine.

Interestingly, it was reported that treatment of Cognitive impairment associated with Alzheimer’s disease (AD) and schizophrenia was associated with α7 nicotinic acetylcholine receptor (α7nAChR) that represented promising therapeutic candidates [190]. As compound **282** (Figure 20) was found, a potent and selective full agonist of the α7 nAChR demonstrated improved plasma stability, brain levels, and efficacy in behavioral cognition models.

On the other side, it was demonstrated that pyrazole **283** proved to be a potent and selective fair pharmacokinetic profile accompanied by efficacy in rodent behavioral cognition models. Compound **284** (Figure 20) was investigated and found as the most potent inhibitor of α7 nAChR with an IC_50_ value of 0.07 µM [191]. Astra Zeneca AB developed diverse series of pyrazole derivatives as positive allosteric modulators (PAMs).

Compound **285** (Figure 20) expressed good activity by inhibiting nicotinic acetylcholine receptors (nAChRs) [192]. The trisubstituted pyrazole **286** (Figure 20) showed unusual activity with a PEC_50_ value of 7.11 (62.68% efficacy) and a PAM type 4 profile [193].

### 4.7. Insecticides and Herbicides

**Figure 21:**
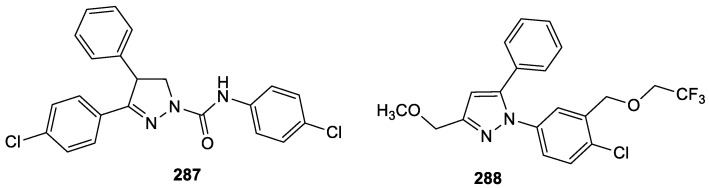
Structures of some pyrazoles **287** and **288** of insecticidal and herbicidal activity.

Synthesized pyrazoline-type insecticides **287** (Figure 21) were achieved and examined the mechanism of action of these compounds based on available electrophysiological, pharmacological, and toxicological information, and they were found to act at neuronal target sites [194].

Compounds 1,5-diarylpyrazole derivative **288** (Figure 21) were prepared and showed noticeable pre- emergent herbicide activities against various kinds of weeds [195].

### 4.8. Anticonvulsant and Antidepressant Activity

**Figure 22:**
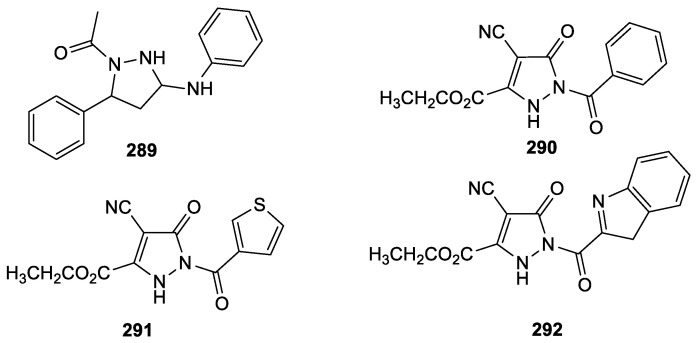
Structures of some pyrazoles **289**–**292** of anticonvulsant and antidepressant activity.

A series of 1-(5-phenyl-3-(phenylamino)pyrazolidin-1-yl)ethanone (**289**) [196] was prepared (Figure 22) and evaluated for anticonvulsant activity against the electric shock-induced convulsion method.

Anti-depressant potency pyrazoles **290**–**292** (Figure 22) showed using tail suspension behavioral despair test and anti-convulsant potency against pentylenetetrazol (PTZ)-induced seizures in mice [197]

### 4.9. Pyrazole as Hypotensive Agents

**Figure 23:**
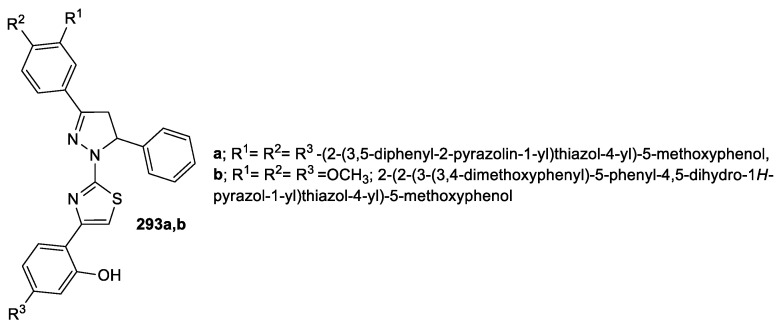
Structures of some pyrazoles **293a**,**b** of Hypotensive activity.

The hypotensive activity of the synthesized 1-(4-arylthiazol-2-yl)-3,5-diaryl-2-pyrazoline derivatives **293a**,**b** (Figure 23) [198] and the compounds were investigated by a tail-cuff method using clonidine as a reference standard. The obtained compounds showed appreciable hypotensive activities.

### 4.10. Anti-HIV

**Figure 24:**
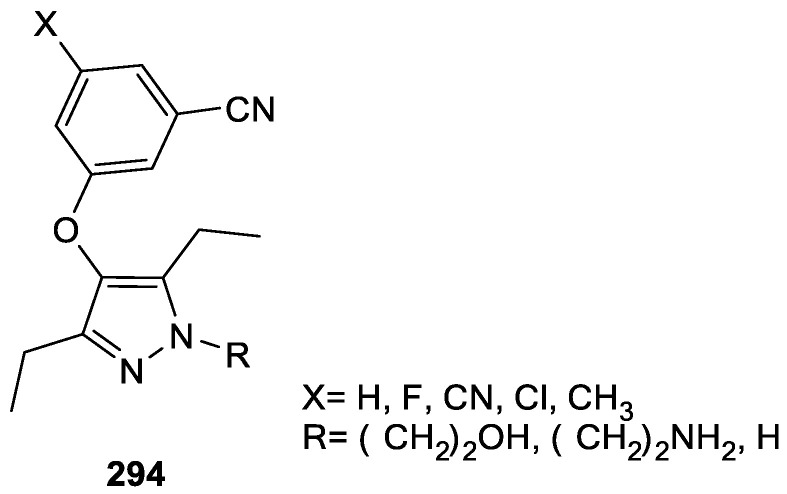
Structure of pyrazole **294** as an anti-HIV agent.

Charles and coworkers constructed 3-cyanophenoxypyrazoles **294** (Figure 24) and investigated it in vitro against HIV. The compounds illustrated excellent anti-HIV affinity with inhibition of wild type RT (IC_50_ = 0.034–0.6 µM) [199].

### 4.11. Hypoglycemic

**Figure 25:**
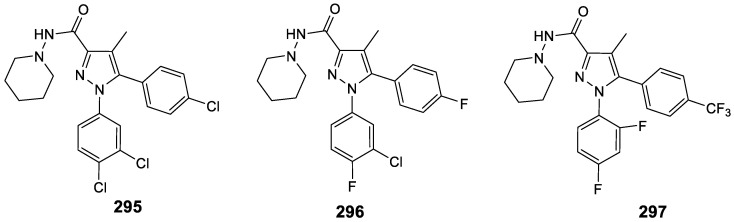
Structures of some pyrazoles **295**–**297**.

1,5-Diaryl pyrazole derivatives **295**–**297** (Figure 25) were synthesized, and the compounds were investigated the biological activity in metabolic disorders, and their hypoglycemic activity in an in vivo model were tested. Interestingly, a high degree of correlation was observed between the predicted p*K*_i_ and hypoglycemic effect after administration. Compounds **295**–**297** showed significant plasma glucose reduction with decreases of 60%, 64%, and 60%, respectively [200].

### 4.12. Anti-Oxidant Activity

**Figure 26:**
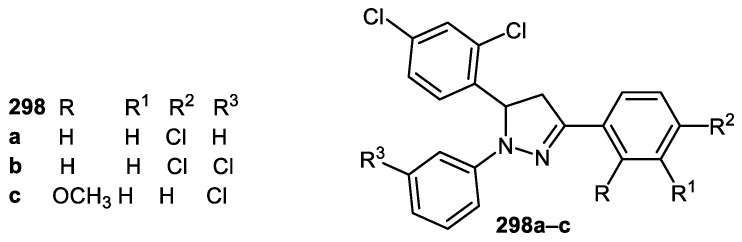
Structures of pyrazoles **298a–c**.

In 2021, Vagish C. B. et al. [201] reported that the synthesized compounds **298** (Figure 26), which revealed modest to good antioxidant activities. The synthesized pyrazoles, **298**, were screened for their antioxidant activity by in vitro DPPH and hydroxyl radical scavenging activity. Assessment result showed that compounds 3-(4-chlorophenyl)-5-(2,4-dichlorophenyl)-1-phenyl-4,5-dihydro-1*H*-pyrazole **298a** revealed % radical scavenging activity

(% I) (20.76–45.14% and 19.46–43.56%), while, 1-(3-chlorophenyl)-3-(4-chlorophenyl)-5-(2,4-dichlorophenyl)-4,5-dihydro-1*H*-pyrazole (**298b**) showed (23.91–46.16% and 20.46–45.07%). Moreover, 1-(3-chlorophenyl)-5-(2,4-dichlorophenyl)-3-(2-methoxyphenyl)-4,5-dihydro-1*H*-pyrazole (**298c**) showed (22.50–42.48% and 20.55–42.80%) show the excellent activities in both DPPH and hydroxyl radical scavenging assay comparable with ascorbic acid and BHA, even with the reported structurally related compounds.

**Scheme 115:**
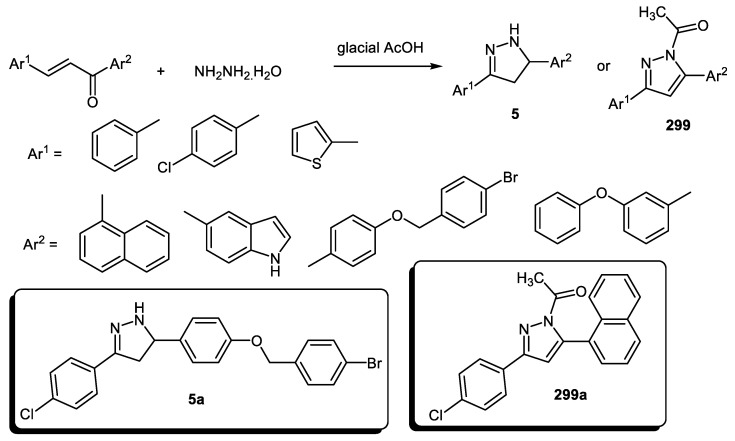
Synthesis of antioxidant pyrazoles **5a** and **299a**.

Mantzanidou et al. [202] evaluated the antioxidant activity of pyrazole derivatives **5a** and **299a**. Compounds **5a** and **299a** were found as the most lipophilic compounds and showed antioxidant activity using the ABTS radical cation (ABTS+) generated through potassium persulfate by oxidation with no participation of an intermediary radical. The synthesis of the pyrazolines and pyrazole derivatives was accomplished via the condensation of substituted suitable chalcones and hydrazine hydrate in absolute ethanol in the presence of drops of glacial acetic acid, as presented in Scheme 115 [202].

## 5. Conclusions

There is a growing body of evidence that pyrazole and its heteroannulated derivatives provide a viable and valuable area for drug discovery. Here, we illustrated an overview of the many efficient, mild, operationally simple, and non-conventional synthetic methods to access a library of highly functionalized pyrazole together with their heteroannulated derivatives. We also shed more light on the broad range of biological activities displayed by these scaffolds that can optimally present a way to capture their intrinsic values. The ability to predict drug-like and lead-like properties along with recent technological advances could be sufficient to revitalize the exploitation of the value of pyrazoles and their heteroannulated derivatives in the quest for new drugs.

Previous studies have shown that the structural modification on the different positions of the basic molecule allows for improving its pharmacological profile, giving it antimicrobial, anticonvulsant, analgesic, anti-inflammatory, anti-viral, anti-malarial, and anti-cancer properties. Recently, researchers have established the design of more potent pyrazole derivatives having a great diversity of biological activity. Afterward, they synthesized the prospective biologically active classes and finally screened the synthesized compounds towards the aim and type of biological activity.

## Figures and Tables

**Table 1:** Synthesis of **11** under different reaction conditions.

Entry	Catalyst	Amount (mol%)	Time (min)	Yield (%)
1	ZnO (Bulk)	5	45	50
2	TiO_2_	5	40	30
3	Al_2_O_3_	5	45	55
4	ZnO (nano)	10	15	95
5	ZnO (nano)	10	25	85
6	ZnO (nano)	20	15	93

**Table 2:** Synthesis of **43** under different reaction conditions.

Entry	Catalyst	Base	Solvent	Yield (%) ^a^
1	CuI	NaOAc	DME	73
2 ^b^	CuI	NaOAc	DME	44
3	CuI	-	DME	trace
4 ^c^	CuI	NaOAc	DME	75
5 ^d^	CuI	NaOAc	DME	75
6	-	NaOAc	DME	0

^a^ Isolated yield. ^b^ Reaction was carried out with 0.2 equiv. of base. ^c^ Reaction was carried out with 2 equiv. of base. ^d^ Reaction was carried out with 1.0 equiv. of base under nitrogen.

**Table 3:** Various trials to obtain best yields of compounds **118** obtained from the reaction in aqueous medium.

Entry	Solvent	Amounts of Catalyst (mol%)	Time (min)	Yield (%)
1	H_2_O	2.5	35	45
2	H_2_O	5	30	53
3	H_2_O	10	30	84
4	H_2_O	15	30	90
5	H_2_O	20	35	81

## Data Availability

The data presented in this study are available on request from the corresponding author.
